# Development of ReaxFF Reactive Force Field for Aqueous Iron–Sulfur Clusters with Applications to Stability and Reactivity in Water

**DOI:** 10.1021/acs.jcim.0c01292

**Published:** 2021-02-22

**Authors:** Evgeny Moerman, David Furman, David J. Wales

**Affiliations:** † Yusuf Hamied Department of Chemistry, University of Cambridge, Lensfield Road,Cambridge CB2 1EW, United Kingdom; ‡ Division of Chemistry, NRCN, P.O. Box 9001, Beer-Sheva 84190, Israel

## Abstract

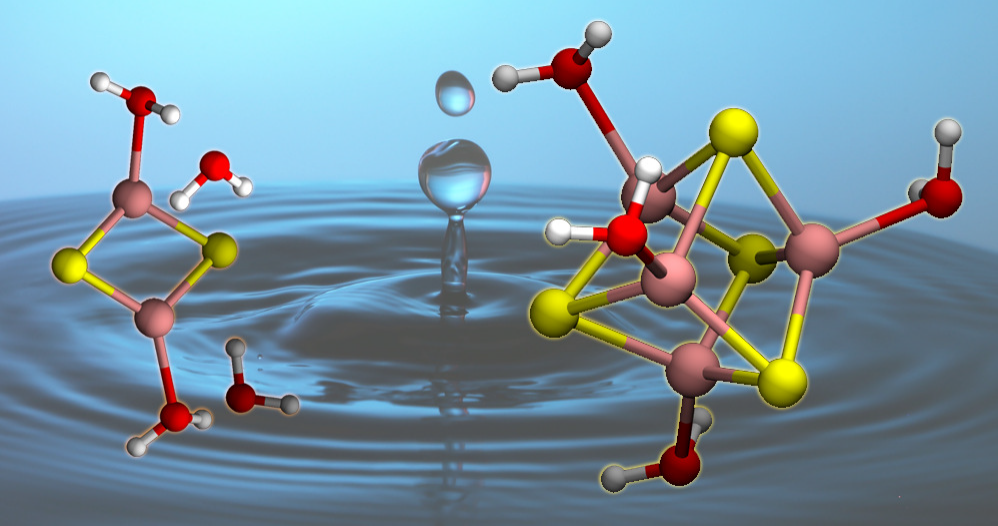

Iron–sulfur clusters serve unique roles in biochemistry, geochemistry, and renewable energy technologies. However, a full theoretical understanding of their structures and properties is still lacking. To facilitate large-scale reactive molecular dynamics simulations of iron–sulfur clusters in aqueous environments, a ReaxFF reactive force field is developed, based on an extensive set of quantum chemical calculations. This force field compares favorably with the reference calculations on gas-phase species and significantly improves on a previous ReaxFF parametrization. We employ the new potential to study the stability and reactivity of iron–sulfur clusters in explicit water with constant-temperature reactive molecular dynamics. The aqueous species exhibit a dynamic, temperature-dependent behavior, in good agreement with previous much more costly ab initio simulations.

## 1 Introduction

Iron–sulfur clusters (Fe_*x*_S_*y*_) are ubiquitous in nature and play important roles in biochemistry and geochemistry.^1^ In biochemical systems, they serve as active sites in FeS proteins, such as ferredoxins, and occur in all organisms, where they are responsible for electron transfer in key biochemical pathways. In some DNA maintenance proteins, Fe–S clusters act as structural components involved in protein complex formation^2^ or supporting catalytic and noncatalytic activities of their host proteins.^3^ Aqueous Fe–S clusters with coordinated H_2_O molecules were first observed by Buffle et al. in lake waters.^4^ Together with ZnS and CuS, they constitute a major fraction of the dissolved metal load in anoxic, sedimentary, freshwater, and deep ocean hydrothermal vents.^5^

Recently, there has been a surge of interest in these systems due to their exceptional chemical properties. As such, they are emerging as novel biomimetic templates,^6^ sustainable batteries,^7^ and catalysts.^8^ For example, a recently synthesized [4Fe–3S] planar cluster, which features an iron center with three bonds to sulfides, has been used to reduce hydrazine, a natural substrate of nitrogenase.^6^ Iron–sulfur clusters are also considered as leading candidates for promoting prebiotic organic synthesis on early Earth.^9^ Central to the theories of the origin of life is the water environment in which the clusters undergo structural transformations,^8^ act as catalytic centers for synthesis of new organic bonds,^10^ and form nucleation sites for minerals such as pyrite and mackinawite.^4^

Despite the widespread applications of iron–sulfur clusters, controversy remains regarding their structure and stability in aqueous environments.^4^ Some of the difficulties are due to the complicated electronic structure of these systems, existence of nonstoichiometric phases, and environment-dependent reactivity.^11,12^ Several recent computational investigations have focused on the static properties of these systems, including the electronic structure and geometry in the gas phase.^13−16^ Other studies utilized nonreactive interatomic potentials and provided important details of their structural properties and the associated bulk phases.^17^ However, analysis of the dynamic nature of the clusters, or the effects of a surrounding aqueous environment, is scarce and limited to *ab initio* approaches.^18,19^

To facilitate large-scale dynamic studies of iron–sulfur clusters in aqueous environments, we report on the development of a new ReaxFF reactive force field designed for Fe_*x*_S_*y*_ clusters that are coordinated to H_2_O molecules. Unlike other potentials, ReaxFF allows us to describe chemical reactions in large systems (10^4^ to 10^6^ atoms) bridging the gap between *ab initio* methods and empirical force fields.^20,21^ As a starting point, we use the Fe–S parameters of Shin et al.,^22^ which include a basic description of Fe–S alloys, while the water parameters were taken from a recent study of biomolecules in solution.^23^ On the basis of new quantum mechanical (QM) calculations, we significantly improve the description of aqueous iron–sulfur clusters. It is noteworthy that Shin et al.’s force field was mainly developed to describe hydrocarbon oxidation using pyrite-covered Cr_2_O_3_ catalysts. As such, its performance on our types of systems is not expected to be accurate, but it still serves as a useful reference target for our force field.

The following clusters were considered: FeS(H_2_O)_3_, FeS_2_(H_2_O)_2_, Fe_2_S_2_(H_2_O)_4_, two structural isomers of Fe_2_S_3_(H_2_O)_3_, Fe_3_S_4_(H_2_O)_4_, and Fe_4_S_4_(H_2_O)_4_. Our choice is motivated by the observations that these clusters form the active site in iron–sulfur proteins. They are small enough to be feasible for accurate DFT treatments, and they also form structural motifs of the respective bulk phases, such as pyrite and mackinawite. In addition, they were extensively studied in the literature, which provides us the possibility to verify our calculations.

The next section provides a description of the computational methods that were used to construct and evaluate the training and validation sets, including the methodology of ReaxFF reactive force field development. Then, results are presented that showcase the performance of the new force field in comparison to the reference QM calculations and with regard to the initial force field of Shin et al. As an application, the subsequent section reports on the stability and reactivity of iron–sulfur clusters in explicit water, as predicted using the new potential. Finally, a summary of results and future possibilities is presented.

## 2 Methods

### 2.1 Quantum Mechanical Calculations

**Figure 1:**
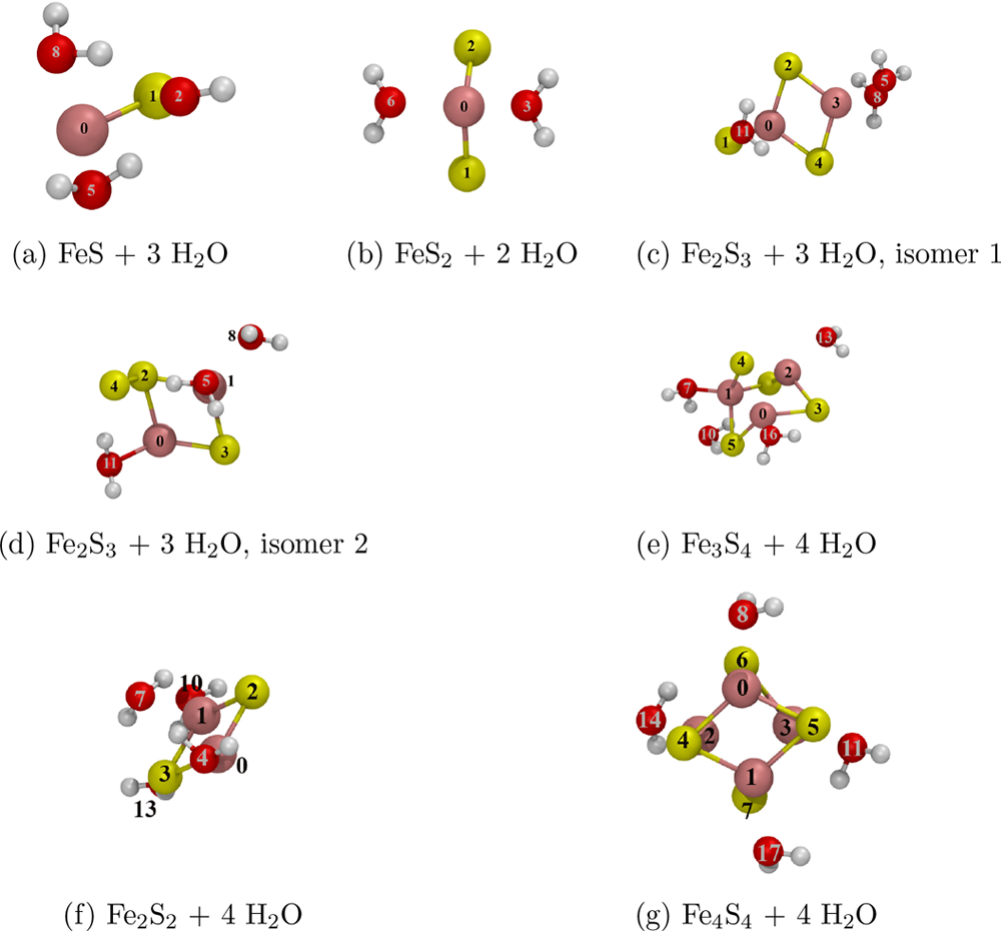
Geometry optimized iron–sulfur clusters at the PBE(D2) level of theory.

The plane-wave DFT code, PWScf, of the Quantum Espresso package (v. 6.1) in conjunction with the ASE interface of Johannes Voss^24^ were employed to obtain optimized structures. The optimizations were performed at the PBE^25^ level with a DFT-D2 dispersion correction.^26^ Ultrasoft GBRV high-throughput pseudopotentials^27^ were used for the plane-wave calculations. A Gaussian smearing width of 0.272 eV (0.02 Ry) was applied to facilitate electronic convergence, which was set to 10^–6^ eV. The clusters were placed in a 20 Å × 20 Å × 20 Å cell and sampled at the gamma point. The calculations were spin-polarized and were accordingly initialized with starting magnetization values of ±0.5 for iron, 0.5 for sulfur, 0.3 for oxygen, and 1.0 for hydrogen. In clusters with more than one iron atom, the sign of the initial magnetic momenta was alternated to facilitate convergence to an antiferromagnetic state.^28−30^ Initial convergence tests showed that an energy cutoff of 550 eV with an 8-fold density cutoff yielded convergence of the energy to ≤0.001 eV per atom. With these settings, the clusters were then geometry optimized until a force of ≤0.005 eV Å^–1^ per atom was reached. The resulting optimized cluster geometries are reported in the Supporting Information. To calculate partial charges, Mulliken population analysis^31^ was performed on the optimized clusters with a spin multiplicity that pertains to the lowest energy, PBE/6-311+G(d,p) level of theory and DFT-D2 dispersion correction^26^ in Gaussian09.^32^Figure 1 presents the optimized cluster geometries together with atomic indices, which will be used to refer to specific atoms throughout the article.

### 2.2 Generation of Training and Validation Sets

The geometry optimized QM structures of FeS(H_2_O)_3_, FeS_2_(H_2_O)_2_, Fe_2_S_2_(H_2_O)_4_, two structural isomers of Fe_2_S_3_(H_2_O)_3_, Fe_3_S_4_(H_2_O)_4_, and Fe_4_S_4_(H_2_O)_4_ were used to construct the training and validation sets. All relevant internal degrees of freedom, including bond lengths, valence angles, torsion angles, and distances between the cluster and surrounding water molecules, were used to generate new nonequilibrium structures by separately scanning each degree of freedom in small increments (typically 0.2 to 0.4 Å for bonds and 10° for angles). Together with the equilibrium geometries and energy differences that were obtained following geometry optimization, the internal coordinate scans and partial charges constituted the main part of the training and validation sets. To allow the force field to describe other key chemical reactions, additional data, including the complete dissociation profiles of molecular sulfur, oxygen, and water, were added to the training set. The training process was iterative, starting with all charge-related parameters. Once the partial charges were accurately reproduced for the equilibrium geometries compared to QM, all other parameters were trained. Apart from the charge related parameters, we have retrained all the H/O/Fe/S combinations of ReaxFF parameters for the bonds, off-diagonal, angles, torsions, and hydrogen bond sections in the force field. The two clusters, Fe_2_S_2_(H_2_O)_4_ and Fe_4_S_4_(H_2_O)_4_, together with all associated properties constituted the validation set for which the transferability of the resulting force field was tested to make sure that other (unseen) structures could also be described satisfactorily.

### 2.3 Training of a ReaxFF Force Field

The training phase aims to find the set of parameters **p** that globally minimize the cost function *C*{**p**}
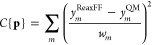
1where the sum adds up the *w*_*m*_-weighted square of the difference between every point of training data as predicted by the force field *y*_*m*_^*ReaxFF*^, and the corresponding quantum mechanical reference value, *y*_*m*_^QM^. To find a putative global minimum, an extended parallel implementation of the RiPSOGM algorithm,^33^ as provided by the open-source code *flocky*, was used.^34^ Inspired by the dynamics of social behavior of bird flocks, the basic algorithm emulates the ability of a set of agents to work as a group in locating promising positions in a given search area.^35^ Here, we use a recently proposed enhanced version of the algorithm that is designed for ReaxFF force field training.^33,34^ Default parameter values were used for the personal and global coefficients (*c*_1_ = 2.0 and *c*_2_ = 2.0, respectively) and initial and final inertia factors (ω_1_ = 0.9 and ω_2_ = 0.4, respectively). Gaussian mutation moves were used to respawn poor-performing members at each step with a scaling factor, γ = 0.1. At the final stages of training, local minimization using the Nelder–Mead algorithm was employed to further relax the positions of every swarm member to a local minimum before the next propagation step for the swarm members. This hybrid approach was found to be more effective for the transformed cost function.^36^ However, due to the high computational burden of evaluating the cost function, this step cannot be performed at every iteration throughout training. A full description of the optimization algorithm can be found in previous work.^33^

### 2.4 ReaxFF Molecular Dynamics

The ReaxFF potential energy of a system is defined by the following terms
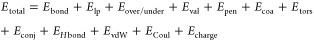
2with tapered^37^ energy contributions from bonding, lone pair electrons, over- and under-coordination, valence angles, penalty for valence angles with two double bonds, three-body conjugation, torsion angles, four-body conjugation, hydrogen bonds, van der Waals (vdW) energy, Coulomb interactions, and self-polarization energy, respectively. All energetic contributions except *E*_vdW_, *E*_Coul_, and *E*_charge_ explicitly depend on the bond order between groups of atoms in a bond, valence angle, or a torsion angle. A full description of all the functional forms can be found in previous publications.^37−39^

The simulation box with periodic boundary conditions (dimensions: 13.0 Å × 16.4 Å × 15.24 Å) for molecular dynamics (MD) simulations consisted of one Fe_2_S_2_ cluster and 78 water molecules with a corresponding water density of 0.99 g mL^–1^. To generate the solvated cluster, the solvation tool of VMD was used.^40^ MD simulations were conducted in LAMMPS^41^ at a constant temperature following standard procedures. In the first stage, energy minimization was carried out to relax the initial system to a root-mean-square gradient (RMSG) of 10^–3^ kcal mol^–1^ Å^–1^. In the next stage, MD simulations at a constant number of atoms, volume, and temperature (NVT ensemble) in the range from 200 to 500 K were performed for 16 ps using a Berendsen thermostat with a coupling constant of 25 fs. The integration time step was set to 0.1 fs in all cases. Analysis was performed on the last 10 ps of the simulation to calculate average bond lengths, angles, and number of hydrogen bonds between the cluster and water molecules.

## 3 Results

### 3.1 Performance on Internal Coordinate Scans

**Figure 2:**
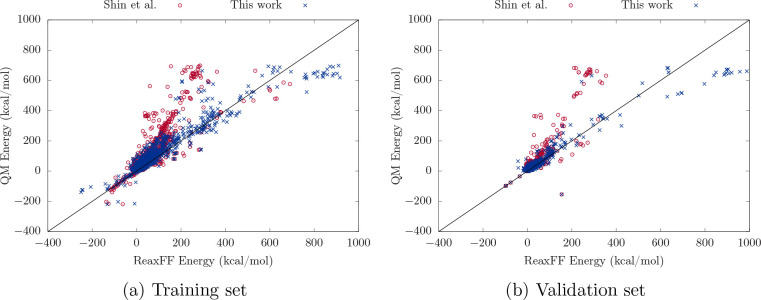
Correlation plots for the energy entries in the training and validation sets comparing the force field trained in this work and the ReaxFF force field of Shin et al.^22^

In the following section, the newly trained force field, denoted ReaxFF-FeS-2020, will be compared to the original force field of Shin et al.,^22^ denoted ReaxFF-Shin-2015. The overall performance with regard to the training and validation sets is summarized in the correlation plots in Figure 2. Each energy entry corresponds to an energy difference between a distorted structure (see Section 2.2) and an equilibrium value.

Figure 2 shows that ReaxFF-FeS-2020 provides a significant improvement compared to ReaxFF-Shin-2015, which exhibits an increasing bias toward underestimating energy differences above 200 kcal mol^–1^ for the training and validation sets. Energy differences between distorted and equilibrium structures of that magnitude correspond to intermediate to highly repulsive distortions, as is shown in the more detailed analysis later. For the extremely repulsive region of the training set of >700 kcal mol^–1^, ReaxFF-FeS-2020 slightly overestimates the energies, but this is usually of no concern, since at very short distances we mainly expect the potential to quickly push the system toward equilibrium geometries. On the other hand, ReaxFF-Shin-2015 exhibits more significant underestimations of the energies in both near-equilibrium and more distorted geometries. Another encouraging observation is that the improved performance of ReaxFF-FeS-2020 is maintained for the validation set, which implies that it should perform well for unseen structures.

**Figure 3:**
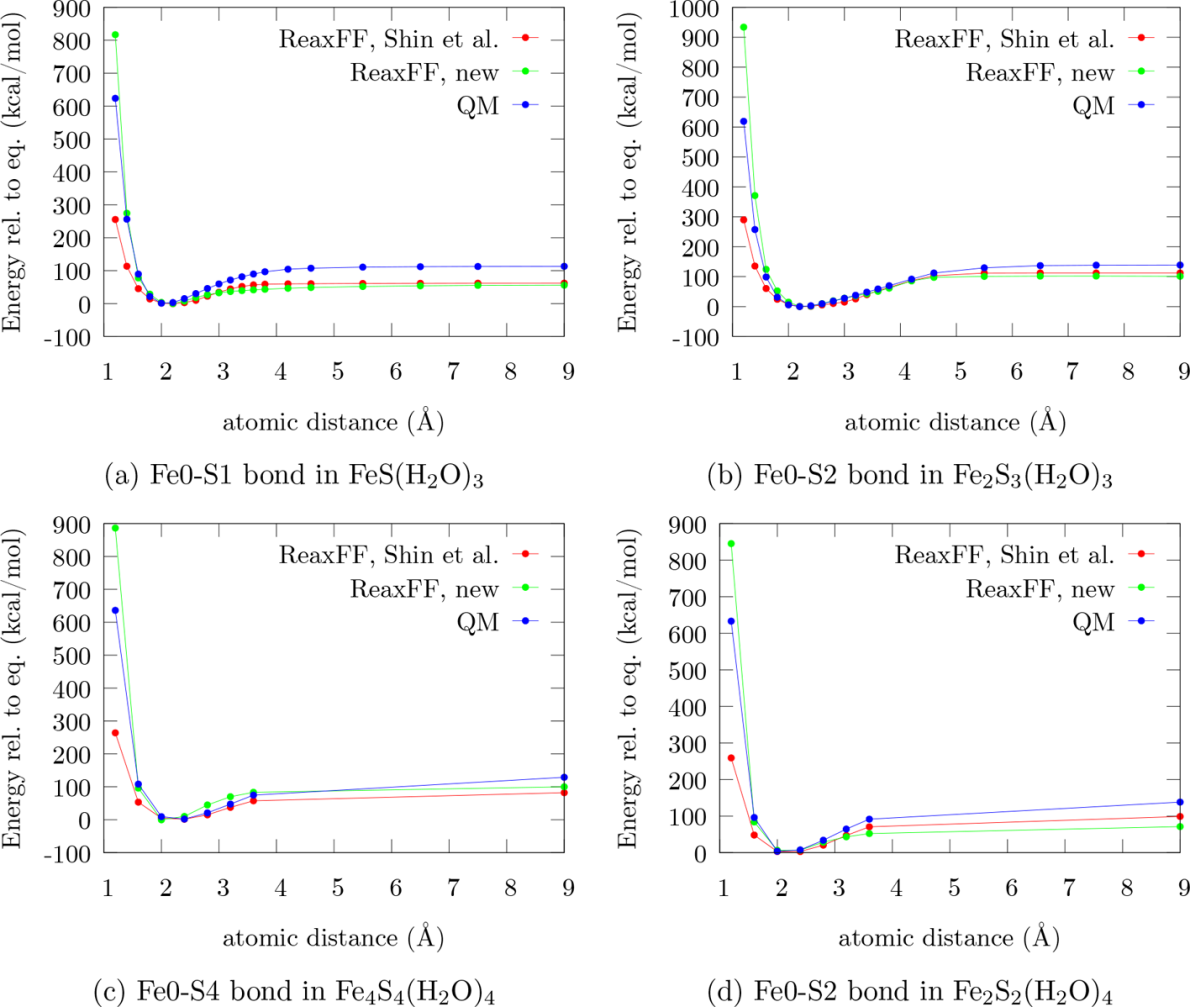
Performance comparison on dissociation curves of the iron–sulfur clusters between ReaxFF-Shin-2015, ReaxFF-FeS-2020, and the QM reference. To uniquely distinguish the atoms involved, the indexing of Figure 1 is used.

**Figure 4:**
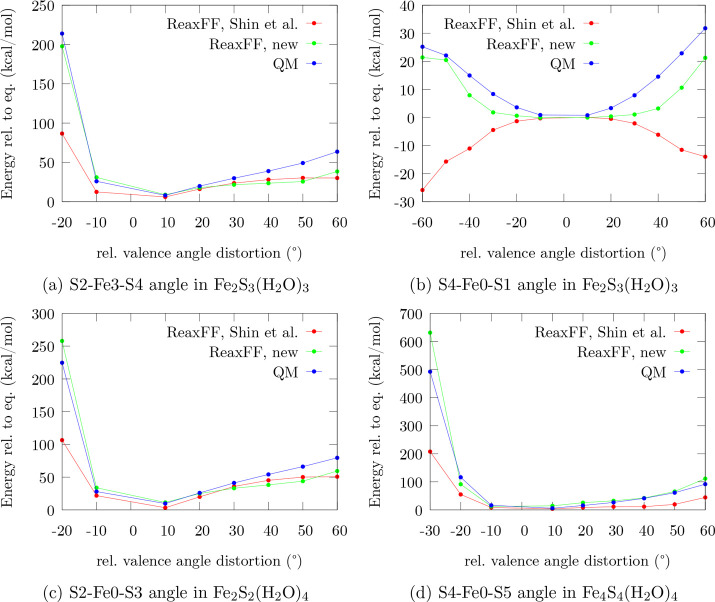
Performance comparison for valence angle profiles of iron–sulfur clusters in the force field by Shin et al., the trained force field, and the QM reference. To uniquely distinguish the atoms involved, the indexing of Figure 1 is used.

**Figure 5:**
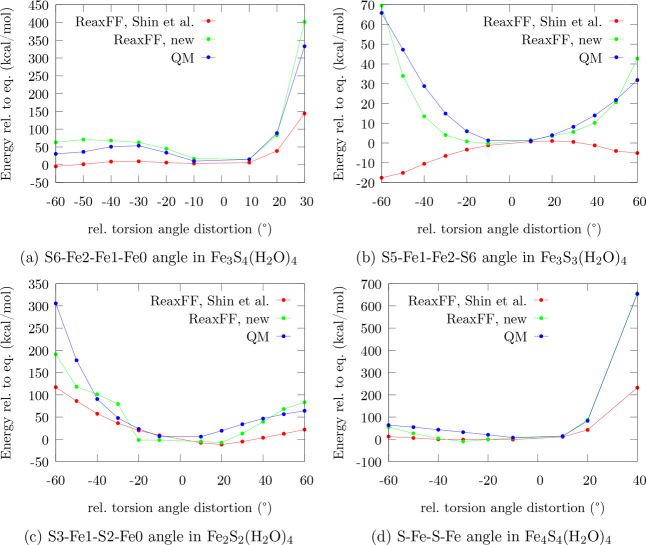
Performance comparison for torsion angle profiles of the iron–sulfur clusters in the force field by Shin et al., the trained force field, and the QM reference. To uniquely distinguish the atoms involved, the indexing of Figure 1 is used.

**Figure 6:**
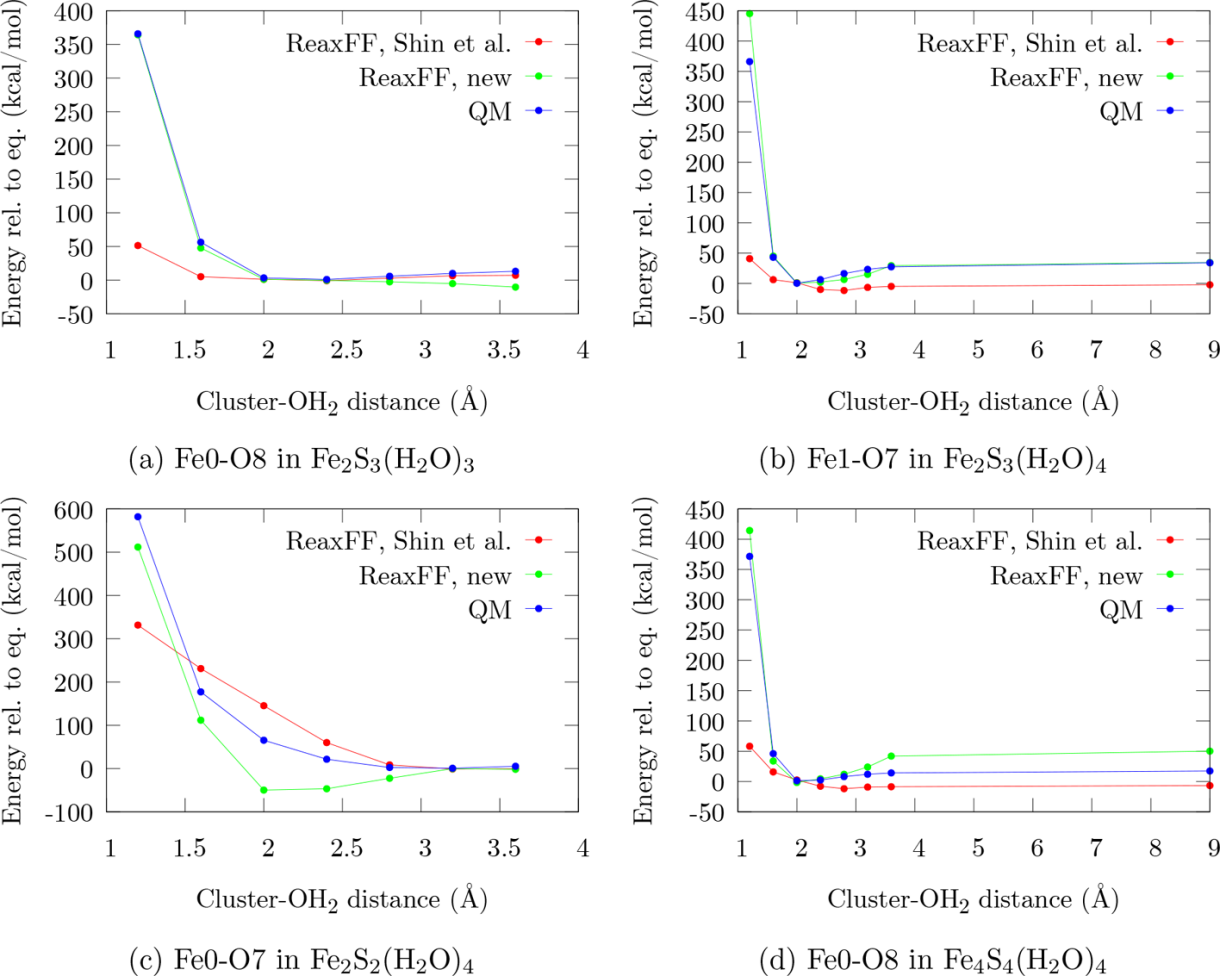
Fe_*x*_S_*y*_–OH_2_ dissociation curves comparing the original force field, the trained force field, and the QM reference. To uniquely distinguish the atoms involved, the indexing of Figure 1 is used. The distance is varied between a cluster atom and the entire H_2_O molecule

To assess the performance of ReaxFF-FeS-2020 in more detail, the energetics of various scans described in Section 2.2 were analyzed. Figures 3–6 present representative examples of dissociation curves for bonds of the iron–sulfur clusters, both from the training set (first row) and validation set (second row).

Figure 3 highlights significant differences between ReaxFF-FeS-2020 and ReaxFF-Shin-2015 in the repulsive region, confirming the trends indicated by the correlation plots in Figure 2. The original force field underestimates the energies, especially in the extremely repulsive regime, by over 50%, while the newly trained force field overestimates it by roughly the same amount. For practical purposes, however, underestimation is significantly more detrimental because it may allow nonphysical small atomic distances. If the highly repulsive nature of two atoms, which are close to each other, is overestimated, this effect will not cause a significant error in the description of the system as long as no extreme conditions are considered where these short bond distances are possible. Except for this difference, the description of the bond dissociation in the equilibrium and the asymptotic regime is fairly similar for the two representations.

From the analogous plots describing the angular distortion of valence angles involving Fe and S in Figure 4, one again observes that the original force field substantially underestimates the repulsive character of close-range interactions between Fe and S atoms. However, including angular distortions that involve the surrounding H_2_O molecules (Fe–S–OH_2_, S–Fe–OH_2_, and H_2_O–Fe–OH_2_), one finds that in some of these cases the repulsive region between O and S is described more accurately by the original parametrization (Supporting Information). Figure 4b shows that in some cases ReaxFF-Shin-2015 describes the energetic profile of angular distortions incorrectly. In the specific case shown in Figure 4b, it predicts the equilibrium angle to be an energetic maximum instead of a minimum. None of these structures were training targets in the Shin et al. force field. Thus, it is unsurprising that some systems are poorly described, and others show an even worse performance. It is likely that such differences originate from an imbalance in the training set, which leads to a biased description.

As for the energy profiles of angular distortions, the description of torsional distortions (Figure 5) in the original force field lacks repulsive character and is sometimes qualitatively wrong, as shown in Figure 5b. In that case, ReaxFF-Shin-2015 predicts the repulsive regions of the distortion profile to be more stable than the QM equilibrium angle, so that the QM equilibrium torsion angle constitutes an energy maximum instead of a minimum. The energy profiles in Figure 5 also suggest that long-range interactions are described more accurately by ReaxFF-FeS-2020, while the previous version mostly underestimates the long-range energy contributions, as highlighted in Figure 5a, where the energy is almost constant in the nonrepulsive region.

To test a key aim of the new force field, namely, the proper description of interactions between iron–sulfur compounds and a potential aqueous environment, one more type of distortion is analyzed in Figure 6. There, the dissociation curve of a water molecule from the cluster atom it is associated with (Fe in most cases) is shown. The QM curves indicate that, depending on the system, the bond strength between iron and water strongly changes between almost zero and over 20 kcal mol^–1^. Also, the difference in curvature of the plots, especially Figure 6a compared with Figure 6b, demonstrates that the covalent or electrostatic nature of the iron–water interaction is also system dependent.

Figure 6 clearly shows that, in contrast to the previously discussed degrees of freedom, in the case of Fe_*x*_S_*y*_–OH_2_ dissociation the performance of ReaxFF-FeS-2020 is substantially different for the training (Figure 6a and b) and validation sets (Figure 6c and d). While the dissociation profiles of the training set are described almost flawlessly by ReaxFF-FeS-2020, the performance for the validation set is less accurate. Nevertheless, the repulsive regime is significantly more accurate than for the original potential. In the near-equilibrium regions, ReaxFF-FeS-2020 again exhibits a much better fit to the QM reference. For the profiles related to the validation set (Figure 6c and d), ReaxFF-FeS-2020 maintains a generally good performance, despite the overestimation of the dissociation energy in Figure 6c. The earlier force field appears to describe the interaction between Fe and H_2_O as purely electrostatic, so that the energy profile can be approximated by a hyperbola, which converges asymptotically to zero.

### 3.2 Equilibrium Cluster Geometries

**Figure 7:**
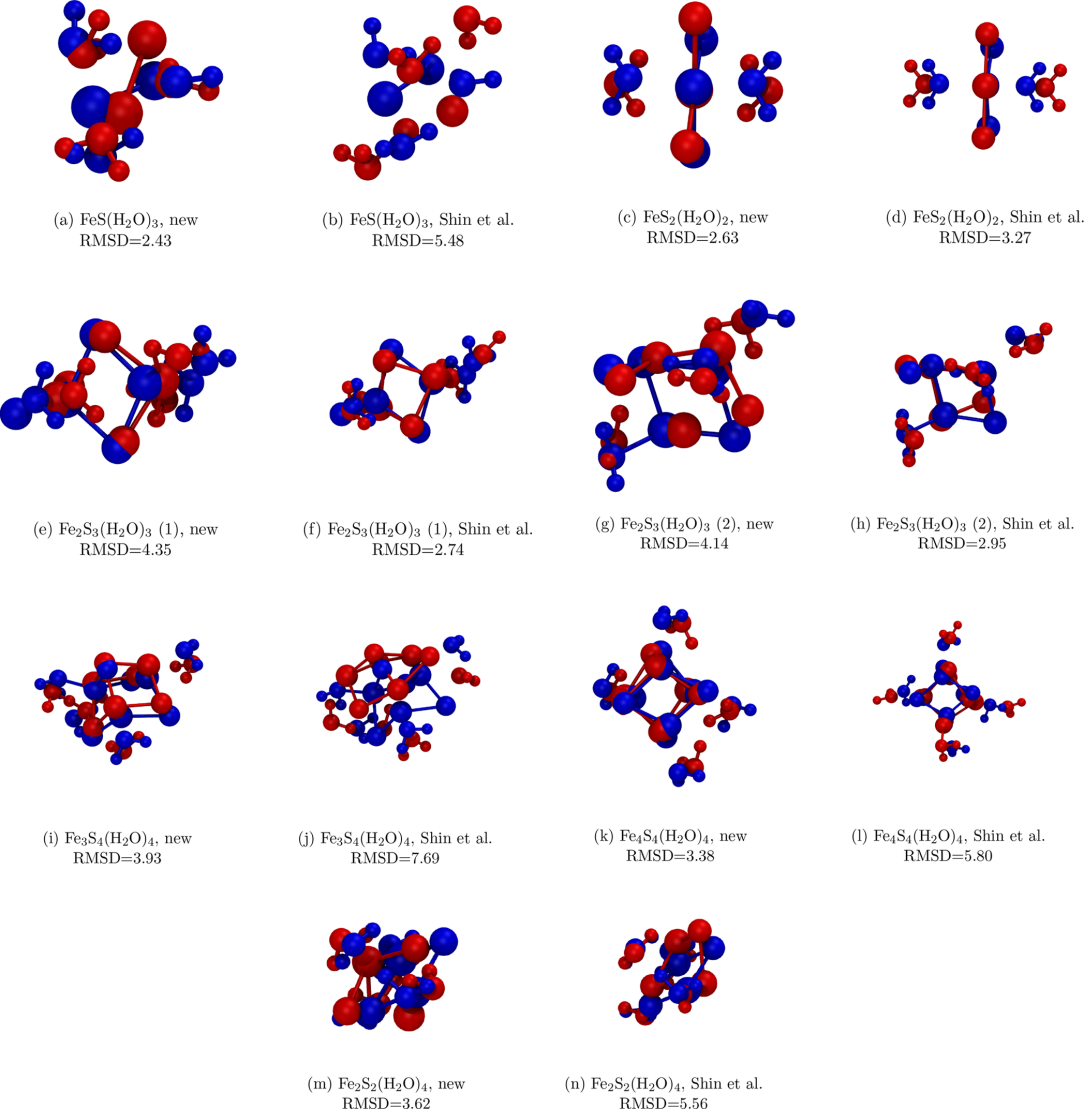
Optimized geometries of the iron–sulfur clusters. The optimal alignment between the structures obtained by the force field (red) and the QM reference (blue) is shown with the root-mean-square deviation (RMSD) in Å. The force field structures were optimized with an RMSG convergence criterion of 10^–4^ kcal mol^–1^ Å^–1^. Visual representations were prepared with VMD.^40^

The focus above was on the energetics of structural distortions, which give direct insight into the topology of the ReaxFF energy landscape, and the energy profiles analyzed in the previous section constitute one-dimensional projections of the potential energy surface (PES). We now investigate the ability of the new force field to predict the structures of iron–sulfur clusters (for both the training and validation sets). The seven iron–sulfur clusters were optimized to an RMSG of <10^–4^ kcal mol^–1^ Å^–1^ with ReaxFF-FeS-2020 and ReaxFF-Shin-2015. The resulting geometries are shown in Figure 7 in superposition with the QM structures.

To analyze the graphical results of Figure 7, the RMSDs of the superpositions are provided as well. These RMSD values refer to the optimally aligned structures with respect to translation, rotation, and permutation-inversion as implemented in the MINPERMDIST routine of OPTIM.^42−44^ The results in Figure 7 show that the ReaxFF-FeS-2020 generally does a better job at predicting the correct cluster structure. Specifically, for four of the seven clusters, it yields substantially better (lower) RMSD values, by up to 50%, and the performance with respect to the training or validation set remains consistent. To refine the comparison, Table 1 presents the averages of absolute bond length, valence angle, and torsion angle deviations for all the clusters. We note that the selected distances and angles in this comparison are the ones that are not rendered redundant due to the symmetry of the system, so that equivalent angles and bond lengths are only counted once in Table 1. Although there are large differences between the degrees of freedom for each cluster, as indicated by the large standard deviations, the averages in Table 1 suggest that ReaxFF-FeS-2020 deviates significantly less from the QM reference in almost every degree of freedom for both the training and validation sets.

**Table 1:** Averages and Standard Deviations of Absolute Errors in Bond Length (Å), Valence Angles (deg), and Torsion angles (deg) for Iron–Sulfur Clusters

	Training set		Validation set	
	new	Shin et al.	new	Shin et al.
bond deviation	1.61 ± 2.72	1.68 ± 2.74	0.37 ± 0.33	0.46 ± 0.47
angular deviation	21.87 ± 22.46	16.41 ± 12.84	9.71 ± 6.89	20.73 ± 15.24
torsional deviation	9.44 ± 15.04	41.77 ± 22.92	6.59 ± 3.78	55.22 ± 91.38

### 3.3 Atomic Partial Charges

**Figure 8:**
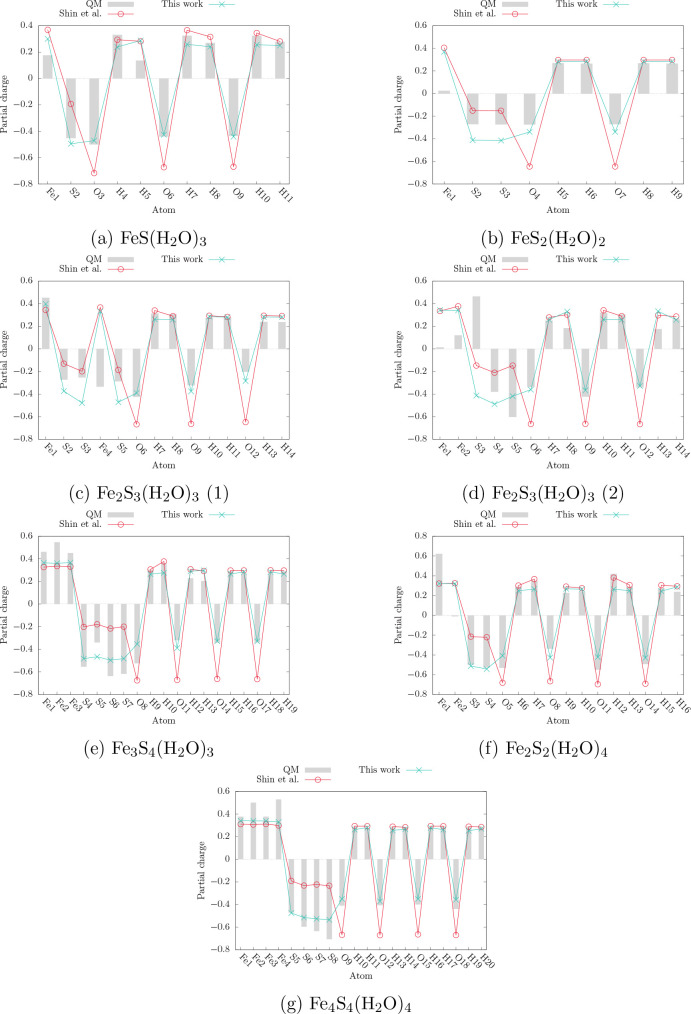
Performance comparison for prediction of partial charges in the training and validation sets. A comparison between the force field trained in this work and the ReaxFF force field of Shin et al.^22^

Finally we assess the atomic partial charges in the clusters. The results are summarized in Figure 8. Both force fields, irrespective of whether the system is part of the training or validation set, predict the Fe atoms to have partial charges between 0.3 and 0.4, which mostly lie slightly below the reference. ReaxFF-FeS-2020 generally provides a slightly improved description compared to the QM reference. For the S atoms, it consistently predicts the atomic charges to be more negative than the previous version and generally closer to the QM reference. As for the oxygen charges, ReaxFF-Shin-2015 reproduces consistently more negative values than ReaxFF-FeS-2020 and the QM reference. In almost every case, ReaxFF-FeS-2020 predicts the oxygen charges more accurately. Finally, the charges of the hydrogen atoms are consistently very similar between the two force fields and are both close to the reference. In two instances, rather strong deviations for both force fields can be attributed to questionable Mulliken charges obtained for the QM reference. The first case is the terminal sulfur atom of the Fe_2_S_3_(H_2_O)_3_ (2) cluster, which is bound to another sulfur atom and is therefore part of the only S–S bond in the training set. This atom, denoted S3 in Figure 8d is predicted to have a significant positive partial QM charge, which is counterintuitive considering the chemical environment. A neutral or negative partial charge, as predicted by both force fields, appears more realistic in this context. In the second case, an iron atom (Fe4) of the Fe_2_S_3_(H_2_O)_3_ cluster features an unphysical negative partial Mulliken charge. Due to the empirical nature of charge population analysis methods (atomic charges are not quantum observables), such discrepancies are not unusual. Overall, the new force field predicts partial charges in better agreement with the QM reference and therefore should provide a better description of the electrostatic environment in iron–sulfur clusters in water.

### 3.4 Stability and Reactivity of Fe_2_S_2_ in Water

To study Fe–S clusters in aqueous environments, a series of constant-temperature MD simulations were carried out in the range from 200 to 500 K. Since all previous calculations involved only gas-phase clusters, it is interesting to analyze the most stable geometry in explicit water. The wide temperature range serves to assess the stability and dynamic nature of the clusters. Experiments have shown that small FeS_(aq)_ clusters are formed rapidly in aqueous solution,^45,46^ but there is still debate over the size and stoichiometry of the solvated species, as well as their stability in water.^4^ In this respect, Fe_2_S_2_ is particularly interesting as it is very similar to the structural unit of mackinawite and constitutes the active center of many proteins. We start the simulation by placing an Fe_2_S_2_ cluster in a water box to observe the formation of a solvated species at different temperatures.

**Figure 9:**
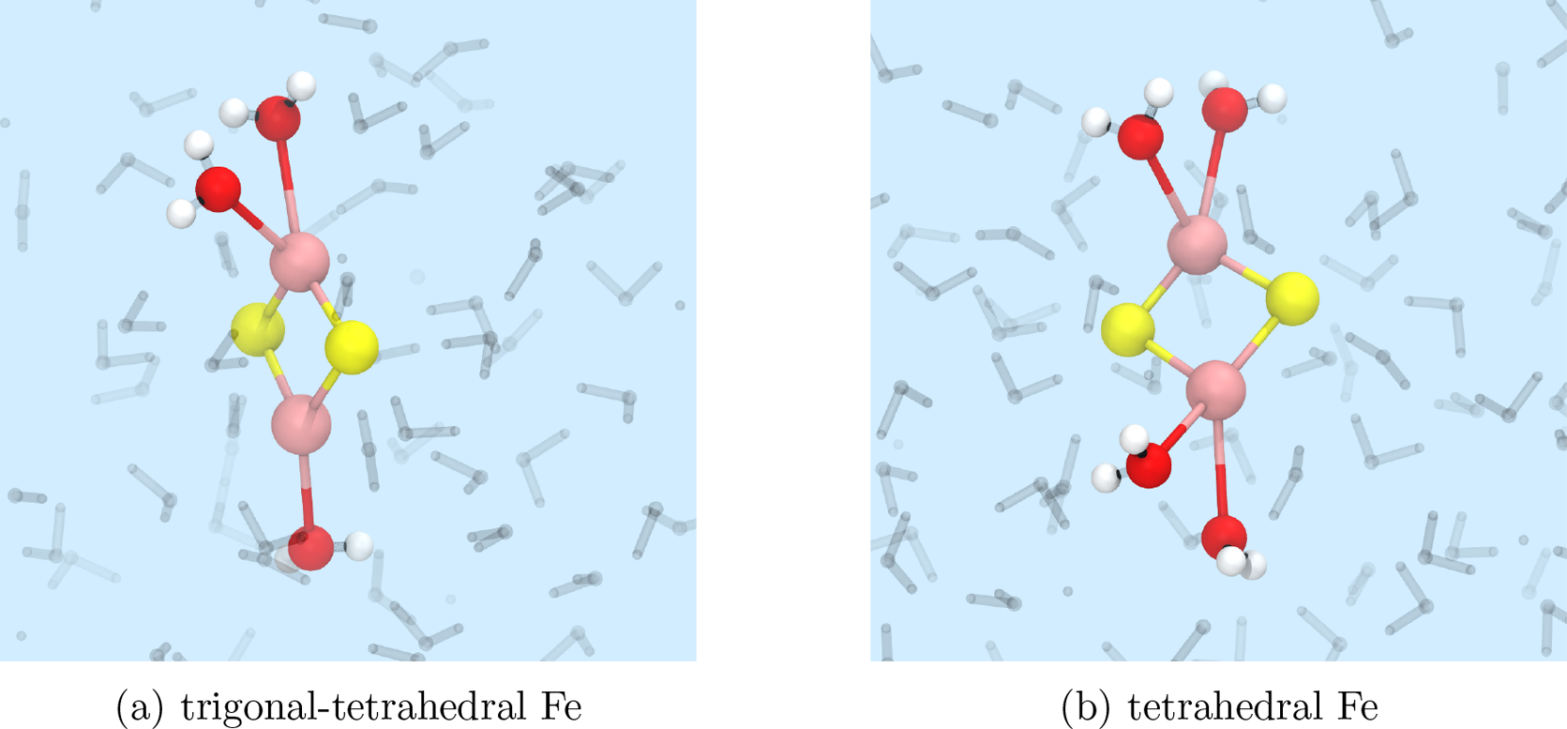
Instantaneous snapshots of the most abundant structures of Fe_2_S_2_ in water at 300 K. Water molecules that are directly coordinated to the cluster are emphasized, while others were made semitransparent: Fe, pink; S, yellow; O, red; and H, white. Visual representations were prepared with VMD.^40^

We find that at all temperatures the solvated species forms almost immediately, but the structure is dynamic. During the simulation, water molecules are observed to bind to the Fe sites with occasional dissociation and rebinding, but two structural motifs are particularly frequent. These most probable structures are presented in Figure 9 and correspond to a simulation at 300 K, although the tetrahedral structure is present as a major species in all temperatures. The results suggest that Fe_2_S_2_(H_2_O)_3_ and Fe_2_S_2_(H_2_O)_4_ are probably the key solvated structures in water below 500 K. Above 400 K, we also observe transient five-coordinated clusters with a bipyramidal geometry, which occur only rarely for lower temperatures. Thus, the picture that emerges from our simulations is that Fe_2_S_2_(H_2_O)_3_ and Fe_2_S_2_(H_2_O)_4_ are preferred at low temperatures, while the formation of Fe_2_S_2_(H_2_O)_5_ is entropically driven and becomes as likely as the tetrahedral geometry at high temperatures (400–500 K). Largely similar observations were recently reported in a DFT MD study of the solvation dynamics of tetrahedral Fe_2_S_2_(H_2_O)_4_ in water.^18^ In that study, the authors reported on the formation of trigonal–tetrahedral, tetrahedral, and bipyramidal tetrahedral clusters at 400 K. The tetrahedral cluster was the least affected by the inclusion of a Hubbard correction (DFT+U) and remained the dominant species, while the identity of the second cluster was dependent on the functional. To further characterize the structural properties of Fe_2_S_2(aq)_, we have calculated average bond distances, angles, and number of hydrogen bonds between the cluster and surrounding water molecules (Table 2).

**Table 2:** Averages and Standard Deviations of Bond Lengths (Å), Cluster Dihedral Angle (deg), and Number of Hydrogen Bonds in Fe_2_S_2(aq)_ at Several Temperatures

*T* (K)	*d* (Fe–S)	*d* (Fe–Fe)	θ (Fe–S–Fe–S)	*n*_HB_
200	2.31 ± 0.03	3.27 ± 0.05	1.08 ± 6.01	9.82 ± 1.27
300	2.29 ± 0.09	3.33 ± 0.10	0.68 ± 6.81	8.29 ± 1.80
400	2.30 ± 0.02	3.27 ± 0.07	3.10 ± 9.73	4.91 ± 1.63
500	2.28 ± 0.07	3.24 ± 0.07	3.38 ± 8.64	4.82 ± 1.66

It can be inferred from Table 2 that the Fe–S skeleton remains fairly stable in this temperature range as its characteristic bonds remain largely unchanged. However, a clear change can be noticed in the dihedral angle, which on average becomes larger. Although the standard deviations are fairly large, making a strict comparison difficult, it is possible to relate the change in the dihedral angle to the equally significant change in the number of hydrogen bonds between the cluster and surrounding water molecules. Here, we have used donor–acceptor cutoff criteria of 3.7 Å and 20°, which encompass both strong and weak hydrogen bond values. A clear trend emerges and suggests that approximately only half of the hydrogen bonds with the cluster remain at higher temperatures. Since hydrogen bonds are known to be directional, the decrease at high temperatures is probably the result of unfavorable contacts. The tetrahedral–bipyramidal species becomes more probable at high temperatures, and the presence of five directly coordinated water molecules makes it more difficult to form new favorable hydrogen bond contacts with the cluster.

Our results are generally in good agreement with a previous^18^ DFT study of Fe_2_S_2_(H_2_O)_4_ at 400 K. It was observed that the average Fe–S and Fe–Fe bond lengths ranged from 2.22 to 2.38 Å and 2.57 to 2.88 Å, respectively, whereas the dihedral angle ranged from 0 to 16.0° with higher values obtained for the Hubbard-corrected PBE. In addition, the number of hydrogen bonds was found to vary between 4.9 and 6.2. We consider such differences consistent with overall good agreement because the force field was not trained on Fe_2_S_2(aq)_; hence, this test can be considered as a true prediction. The largest deviations arise from the dihedral angle, which is expected, since dihedral interactions are significantly harder to reproduce correctly due to their many-body nature.

## 4 Summary

On the basis of new quantum chemical calculations on several Fe–S clusters with coordinated water molecules, a new ReaxFF reactive force field parametrization was developed. The training process involved scans of bonds, valence angles, dihedrals, and Fe–S–water intermolecular distances to cover most of the relevant pathways for Fe–S clusters in aqueous environments. The construction of a separate validation set was used to avoid overfitting the force field on the training data, thus retaining enough transferability to describe similar systems. The new force field was shown to outperform a previous parametrization that included Fe–S in its training set, although it was not specifically designed for Fe–S clusters. The new force field was then utilized in reactive molecular dynamics simulations in explicit water to test the parametrization and to provide insights into the structure and stability of Fe–S clusters in water. We found that the most stable geometry of Fe_2_S_2(aq)_ includes tetrahedral Fe sites with four coordinated water molecules, Fe_2_S_2_(H_2_O)_4_. A three-coordinated trigonal structure is also present but is less stable. Above 400 K, the trigonal structure is no longer observed. Instead, the most favorable structures are the tetrahedral and a five-coordinated tetrahedral–bipyramidal structure, Fe_2_S_2_(H_2_O)_5_. The current force field is provided in the Supporting Information and serves as the first step toward the development of a combined inorganic–organic force field for biochemical systems. Since it is augmented with C/H/O/N parameters from a previously developed force field for biomolecules, it can be used in simulations of organic systems. In the future, we plan to expand the current force field to describe catalytic reactions of biomolecules facilitated by pyrite clusters in aqueous environments. Such efforts are currently ongoing and will be reported in a future study.
