# Supplementary material for: Development of ReaxFF Reactive Force Field for Aqueous Iron–Sulfur Clusters with Applications to Stability and Reactivity in Water
Source: J Chem Inf Model. 2021 Feb 22;61(3):1204–14. doi: 10.1021/acs.jcim.0c01292 (PMC8028049; doi:10.1021/acs.jcim.0c01292)
Supplement: Supplementary file 1 — ci0c01292_si_001.pdf [file ci0c01292_si_001.pdf]

Development of ReaxFF Reactive Force Field for Aqueous Iron-sulfur Clusters with Applications to Stability and Reactivity in Water — Supporting Information

Evgeny Moerman,[†] David Furman,[*,‡,†] and David J. Wales[*,†]

[†]*Department of Chemistry, University of Cambridge, Lensfield Road, Cambridge CB2 1EW, United Kingdom*

[‡]*Division of Chemistry, NRCN, P.O. Box 9001, Beer-Sheva 84190, Israel*

E-mail: df398@cam.ac.uk; dw34@cam.ac.uk

1 QM structures used in training

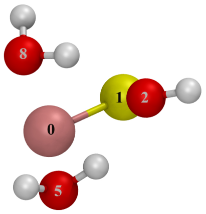

(a) $FeS + 3\ H_2O$

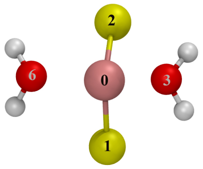

(b) $FeS_2 + 2\ H_2O$

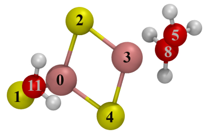

(c) $Fe_2S_3 + 3\ H_2O$, isomer 1

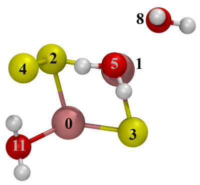

(d) $Fe_2S_3 + 3\ H_2O$, isomer 2

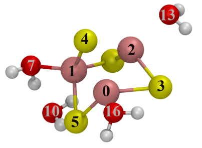

(e) $Fe_3S_4 + 4\ H_2O$

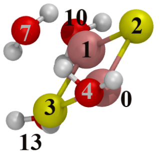

(f) $Fe_2S_2 + 4\ H_2O$

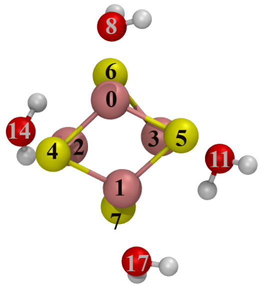

(g) $Fe_4S_4 + 4\ H_2O$

Figure S1: Optimized iron-sulfur clusters on PBE(D2) level of theory

2 Energy profiles of cluster distortions

The different degrees of freedom (atomic distances, valence angles and torsion angles) are addressed using the indexation scheme of figure 4.

2.1 FeS + $3H_2O$

2.1.1 Dissociation profiles

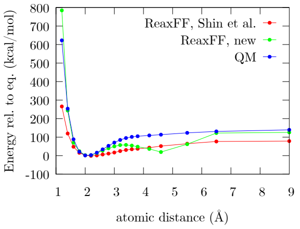

(a) S1-Fe0

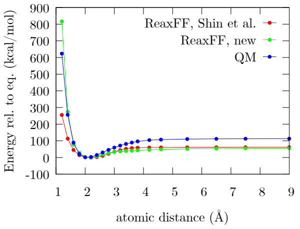

(b) Fe0-S1

2.1.2 Angular energy profiles

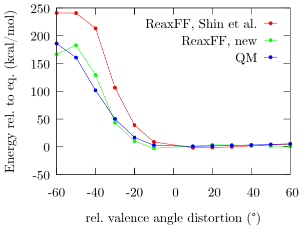

(a) S1-Fe0-O5

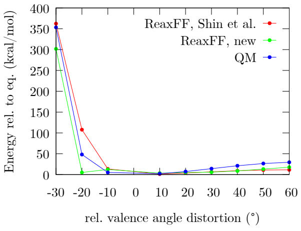

(b) O8-Fe0-O2

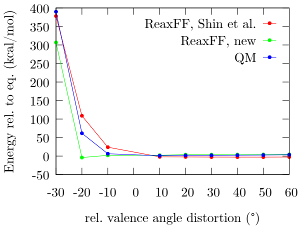

(c) O2-Fe0-O5

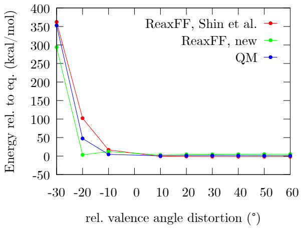

(d) O2-Fe0-O8

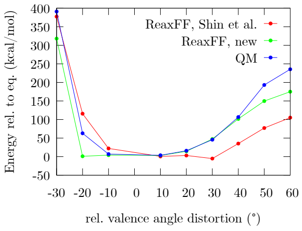

(e) O5-Fe0-O2

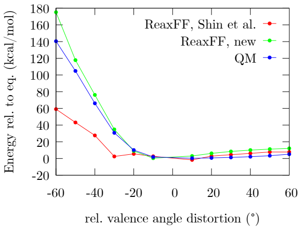

(f) O5-Fe0-S1

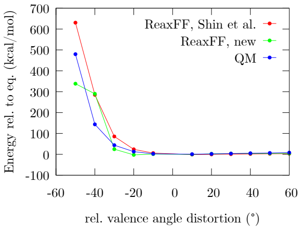

(g) O8-Fe0-O5

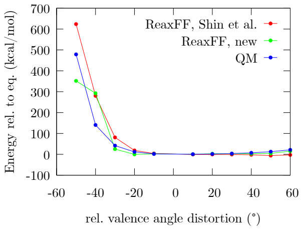

(h) O5-Fe0-O8

2.1.3 Cluster-H_2O dissociation profiles

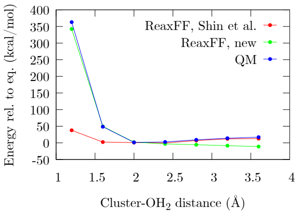

(a) Fe0-O5

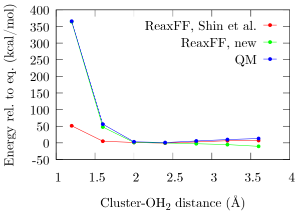

(b) Fe0-O8

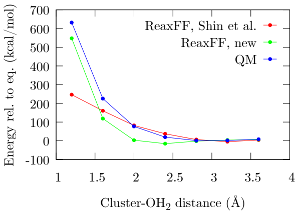

(c) Fe0-O2

2.2 $FeS_2 + 2H_2O$

2.2.1 Dissociation profiles

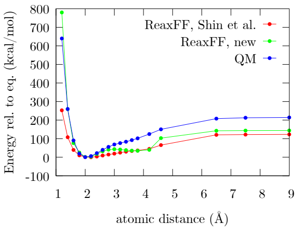

(a) S1-Fe0

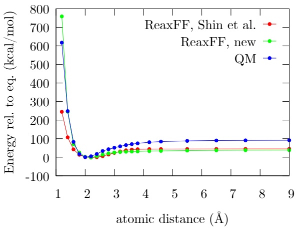

(b) Fe0-S1

2.2.2 Angular energy profiles

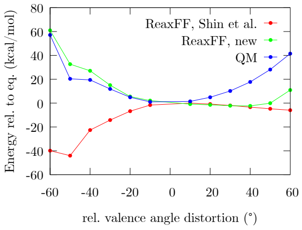

(a) S1-Fe0-S2

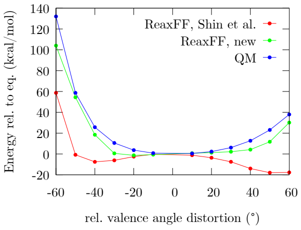

(b) O3-Fe0-S1

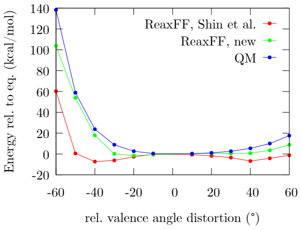

(c) S1-Fe0-O3

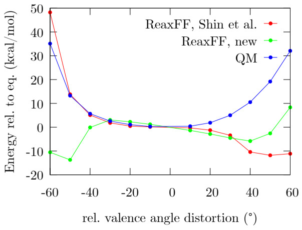

(d) O3-Fe0-O6

2.2.3 Cluster-H_2O dissociation profiles

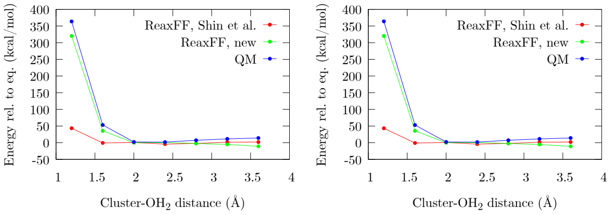

(a) Fe0-O6 (b) Fe0-O3

2.3 Fe_2S_3 + $3H_2O$ (1)

2.3.1 Dissociation profiles

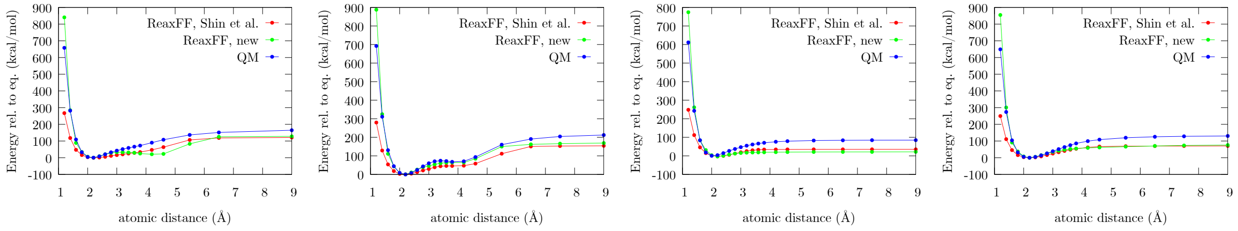

(a) S2-Fe3 (b) S2-Fe0 (c) Fe0-S1 (d) Fe0-S2

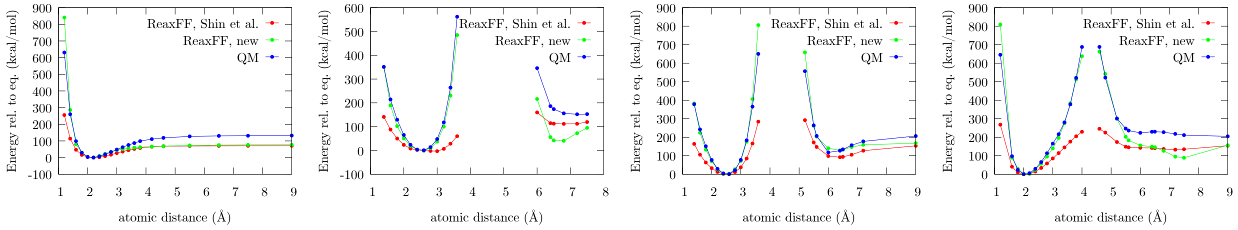

(e) Fe3-S2 (f) Fe0-Fe3 (g) Fe3-Fe0 (h) S1-Fe0

2.3.2 Angular energy profiles

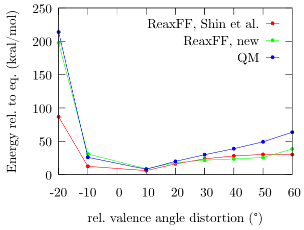

(a) S2-Fe3-S4

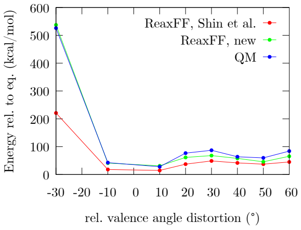

(b) Fe3-S2-Fe0

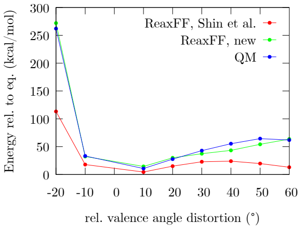

(c) S2-Fe0-S4

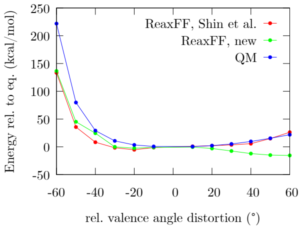

(d) S2-Fe3-O8

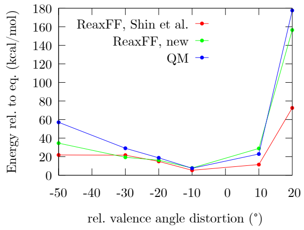

(e) O5-Fe3-S2

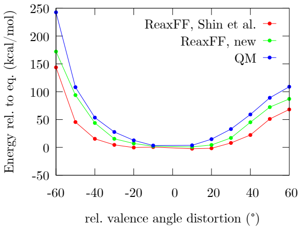

(f) O8-Fe3-S2

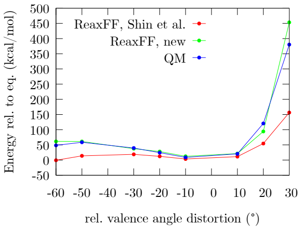

(g) S1-Fe0-S4

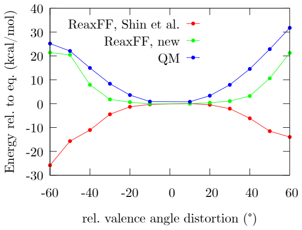

(h) S4-Fe0-S1

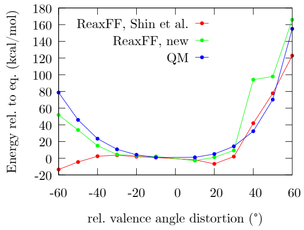

(i) S1-Fe0-O11

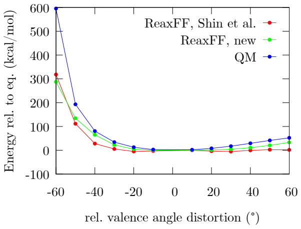

(j) O11-Fe0-S2

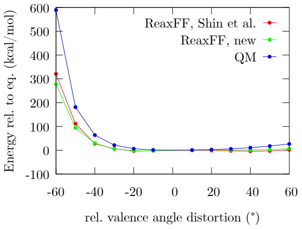

(k) S2-Fe0-O11

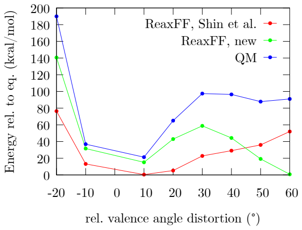

(l) Fe0-S2-Fe3

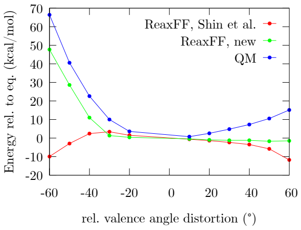

(m) O11-Fe0-S1

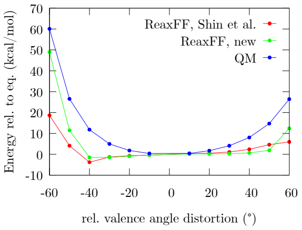

(n) S2-Fe3-O5

2.3.3 Torsional energy profiles

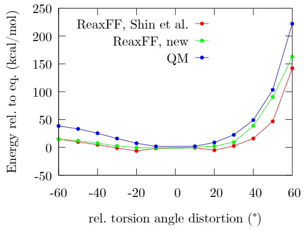

(a) Fe0-S2-Fe3-S4

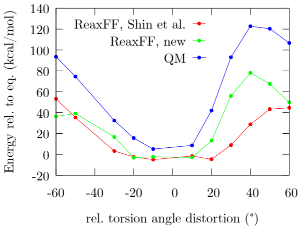

(b) S1-Fe0-S2-Fe3

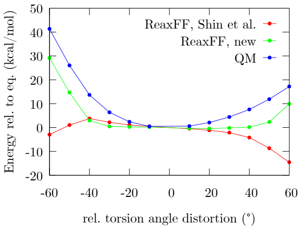

(c) Fe3-S2-Fe0-S1

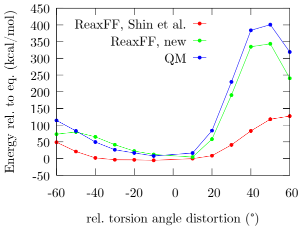

(d) S4-Fe3-S2-Fe0

2.3.4 Cluster-H_2O

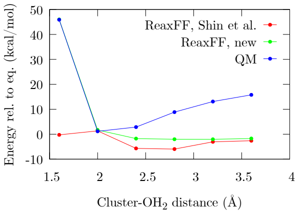

(a) Fe3-O5

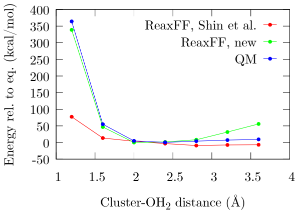

(b) Fe0-O11

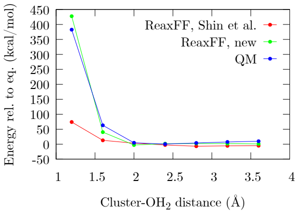

(c) Fe3-O8

2.4 Fe_2S_3 + $3H_2O$ (2)

2.4.1 Dissociation profiles

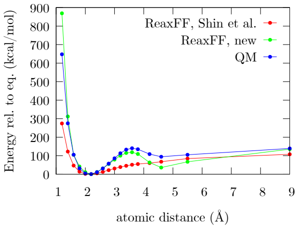

(a) S3-Fe0

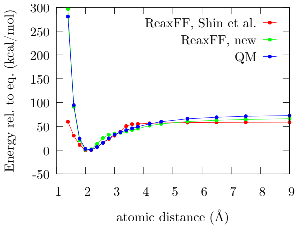

(b) S2-S4

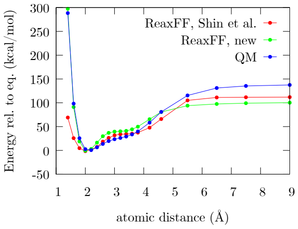

(c) S4-S2

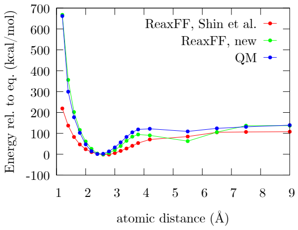

(d) Fe1-Fe0

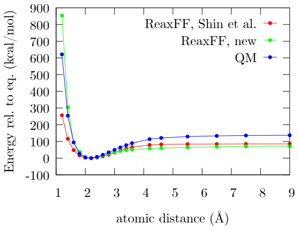

(e) Fe0-S3

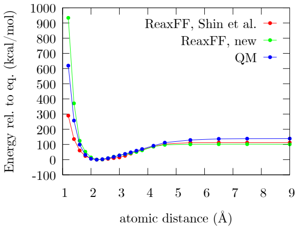

(f) Fe0-S2

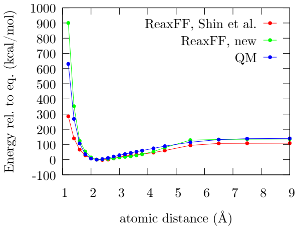

(g) S2-Fe0

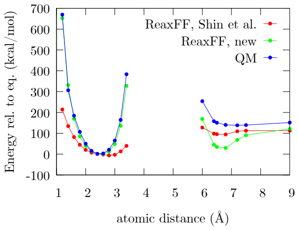

(h) Fe0-Fe1

2.4.2 Angular energy profiles

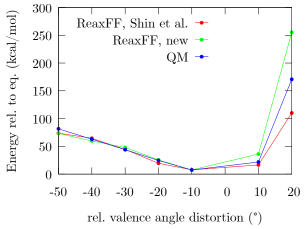

(a) O8-Fe1-S2

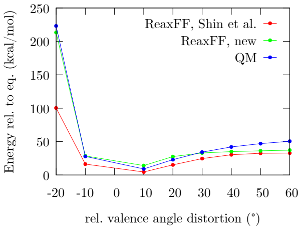

(b) S2-Fe0-S3

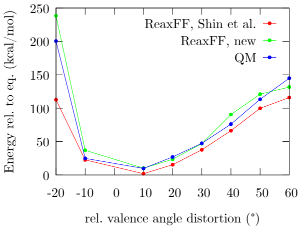

(c) S3-Fe0-S2

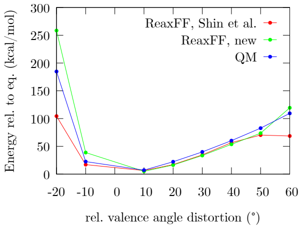

(d) S3-Fe1-S2

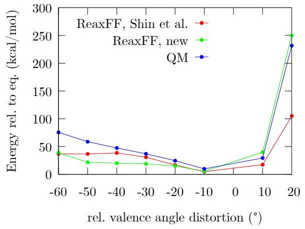

(e) O8-Fe1-S3

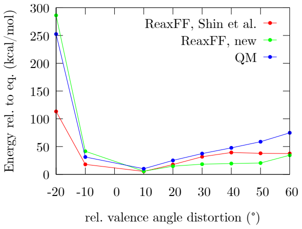

(f) S2-Fe1-S3

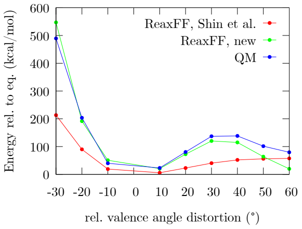

(g) Fe1-S2-Fe0

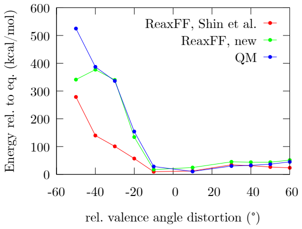

(h) O5-Fe1-S2

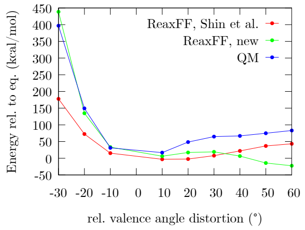

(i) Fe0-S3-Fe1

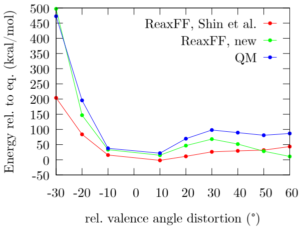

(j) Fe0-S2-Fe1

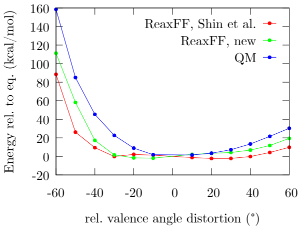

(k) O5-Fe1-S3

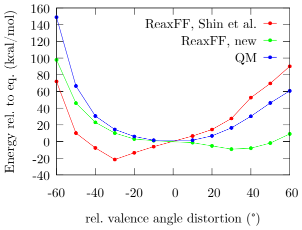

(l) S3-Fe1-O5

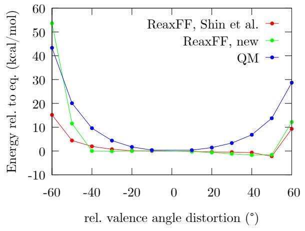

(m) S2-Fe1-O8

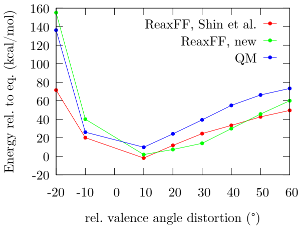

(n) Fe1-S3-Fe0

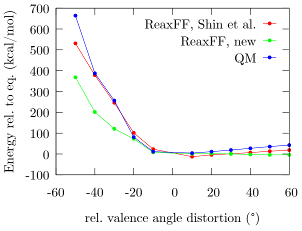

(o) S2-Fe1-O5

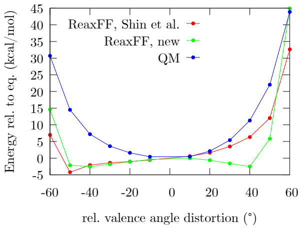

(p) S3-Fe1-O8

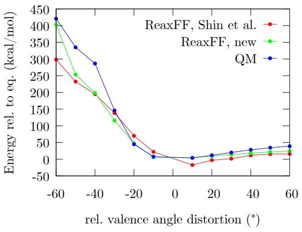

(q) Fe1-S2-S4

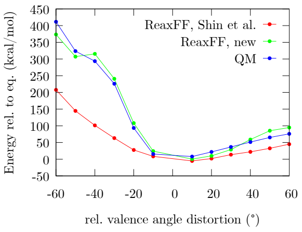

(r) S4-S2-Fe1

2.4.3 Torsional energy profiles

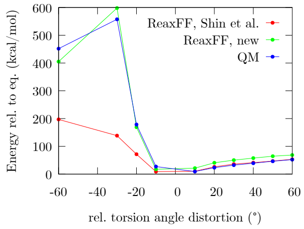

(a) Fe1-S3-Fe0-S2

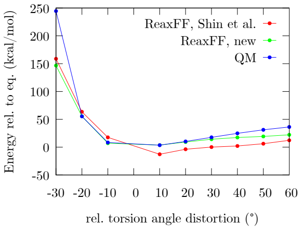

(b) S3-Fe1-S2-S4

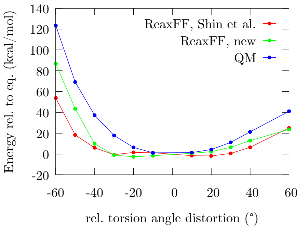

(c) Fe0-S2-Fe1-S3

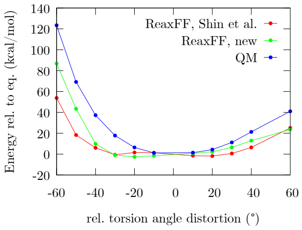

(d) S4-S2-Fe1-S3

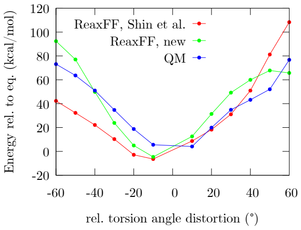

(e) S3-Fe1-S2-Fe0

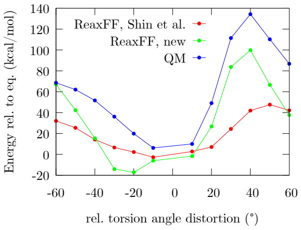

(f) S2-Fe0-S3-Fe1

2.4.4 Cluster-H_2O dissociation profiles

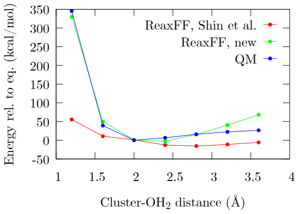

(a) Fe0-O11

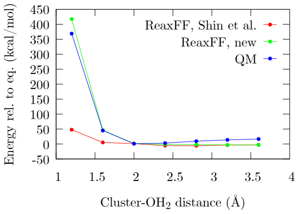

(b) Fe1-O8

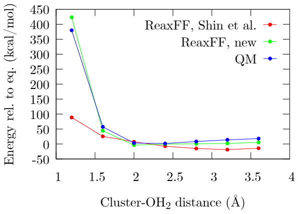

(c) Fe1-O5

2.5 $Fe_3S_4 + 4H_2O$

2.5.1 Dissociation profiles

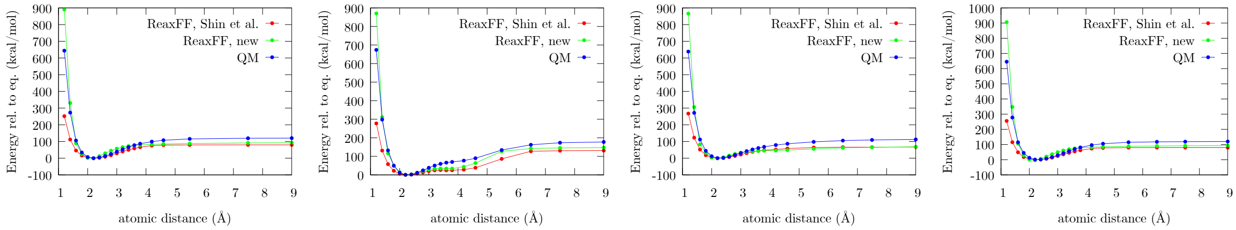

(a) Fe1-S4 (b) S3-Fe0 (c) Fe0-S3 (d) Fe0-S4

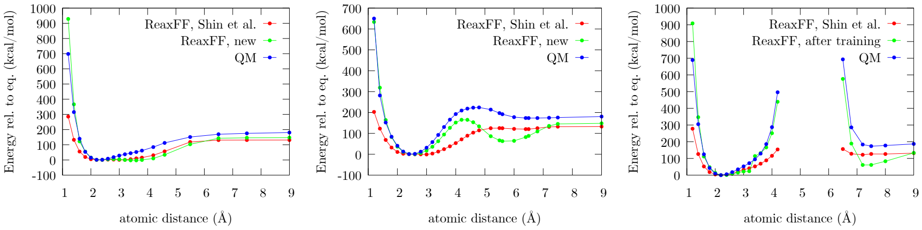

(e) S4-Fe0 (f) Fe0-S2 (g) S4-Fe1

2.5.2 Angular energy profiles

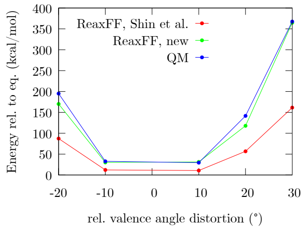

(a) S3-Fe0-Fe2

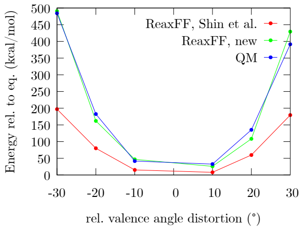

(b) Fe1-Fe0-Fe2

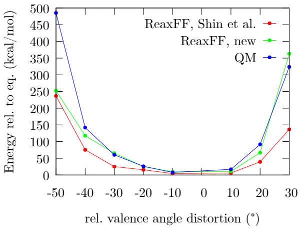

(c) O13-Fe2-S3

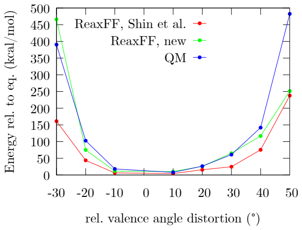

(d) Fe1-Fe0-S3

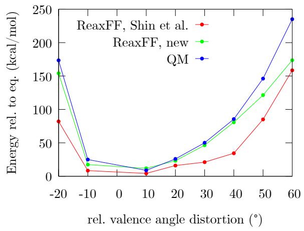

(e) Fe2-Fe0-S3

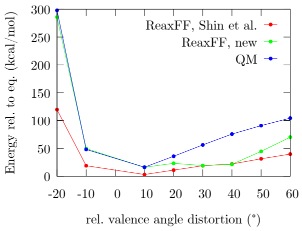

(f) S3-Fe0-Fe1

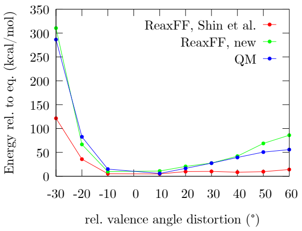

(g) S3-Fe0-S4

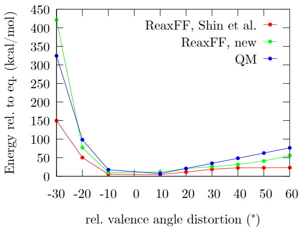

(h) S4-Fe0-S3

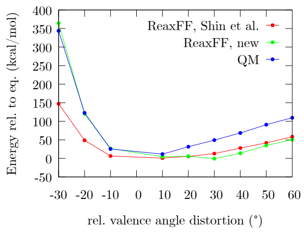

(i) Fe0-S4-Fe2

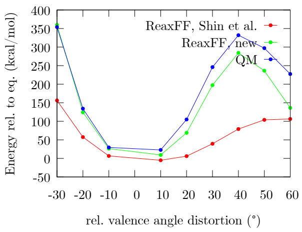

(j) Fe0-S3-Fe2

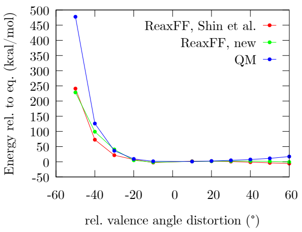

(k) S3-Fe2-O13

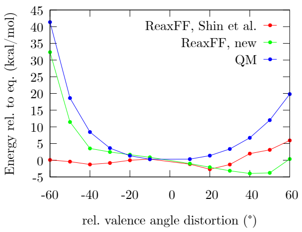

(l) S4-Fe2-O13

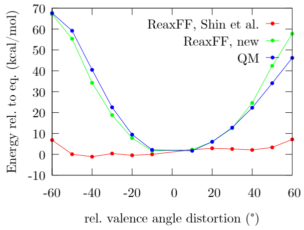

(m) S3-Fe0-S5

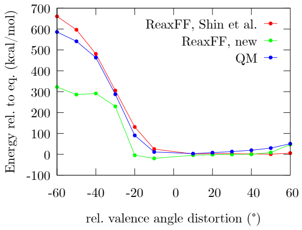

(n) Fe1-Fe0-O17

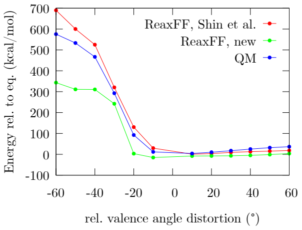

(o) O7-Fe1-O10

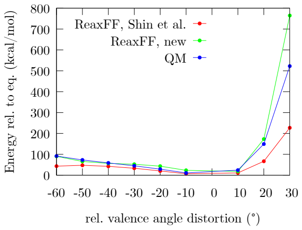

(p) O13-Fe2-S4

2.5.3 Torsional energy profiles

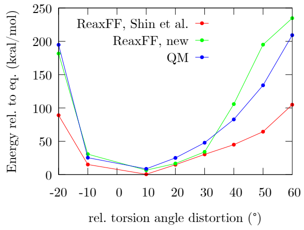

(a) Fe0-S5-Fe0-S6

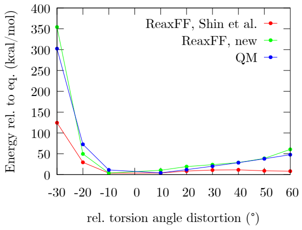

(b) S6-Fe2-Fe1-S4

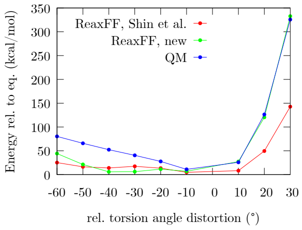

(c) S6-Fe1-S4-Fe0

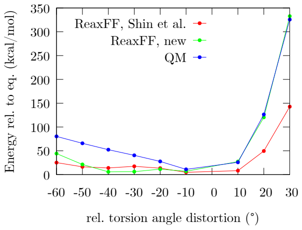

(d) S5-Fe1-S4-Fe0

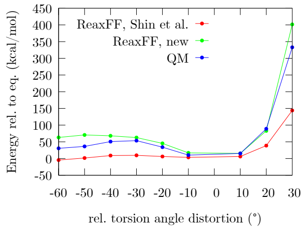

(e) S6-Fe2-Fe1-Fe0

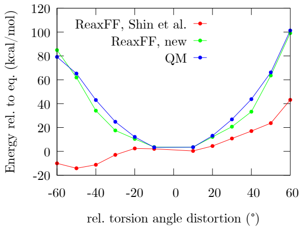

(f) Fe0-S4-Fe1-S5

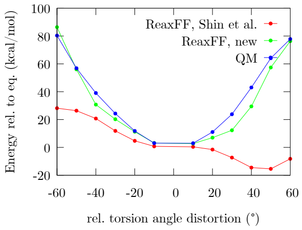

(g) Fe0-S4-Fe1-S6

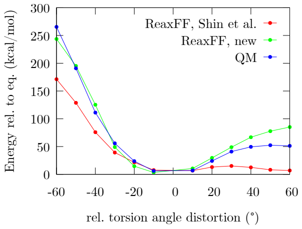

(h) S6-Fe2-Fe1-S5

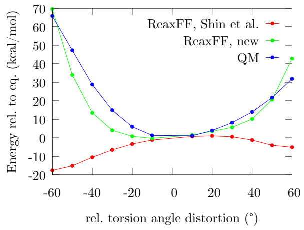

(i) Fe0-Fe1-Fe2-S6

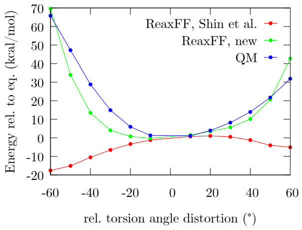

(j) S4-Fe1-Fe2-S6

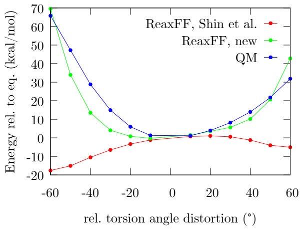

(k) S5-Fe1-Fe2-S6

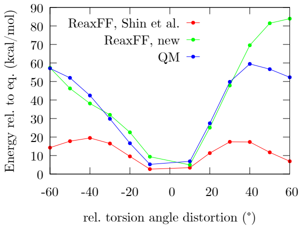

(l) S5-Fe0-Fe2-S6

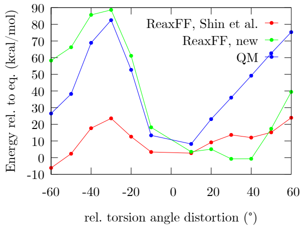

(m) S6-Fe1-S5-Fe0

2.5.4 Cluster-H_2O dissociation profiles

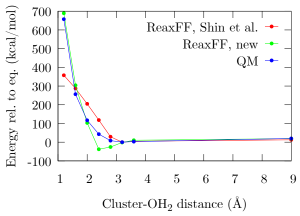

(a) Fe1-O10

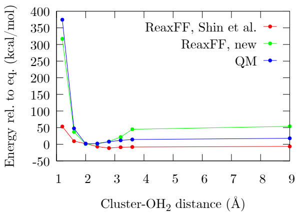

(b) Fe0-S16

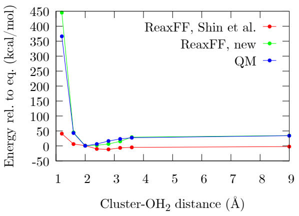

(c) Fe1-O7

2.6 $Fe_2S_2 + 4H_2O$

2.6.1 Dissociation profiles

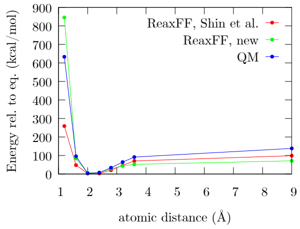

(a) Fe0-S2

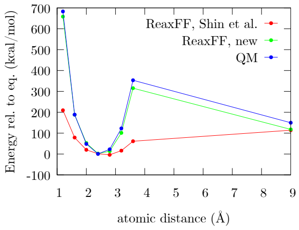

(b) Fe1-Fe0

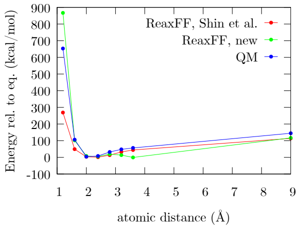

(c) S2-Fe0

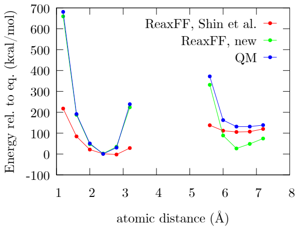

(d) Fe0-Fe1

2.6.2 Angular energy profiles

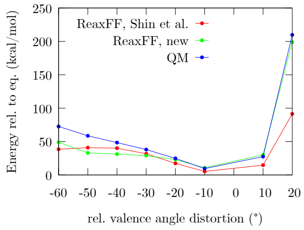

(a) O10-Fe1-S2

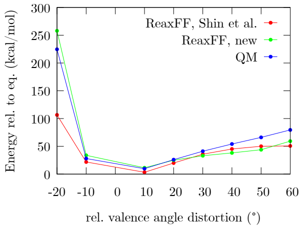

(b) S2-Fe0-S3

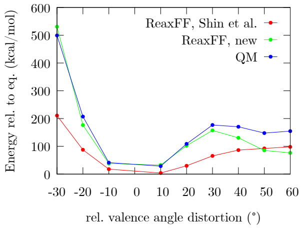

(c) Fe0-S3-Fe1

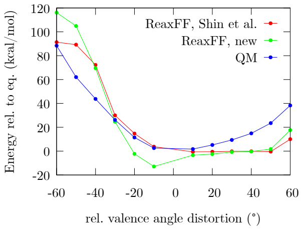

(d) S3-Fe0-O4

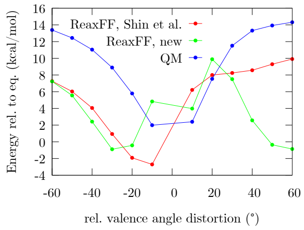

(e) S2-Fe1-O13

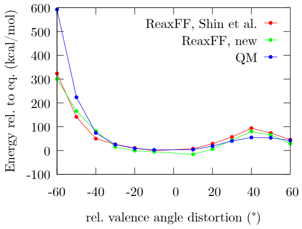

(f) S3-Fe0-O7

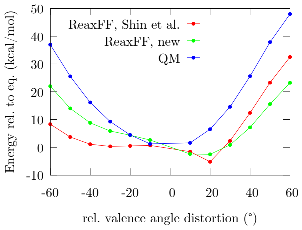

(g) O13-Fe1-S2

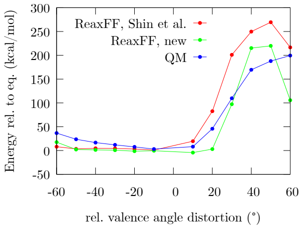

(h) S2-Fe1-O10

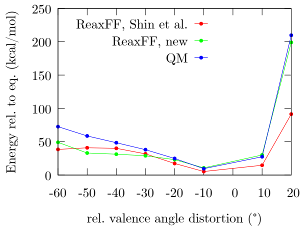

(i) Fe1-Fe0-O12

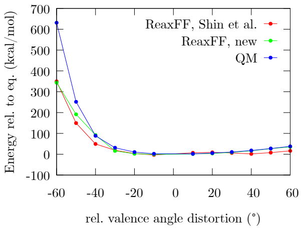

(j) O7-Fe0-S3

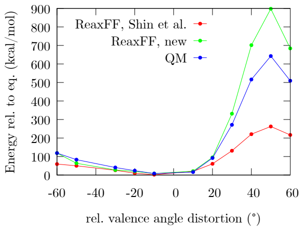

(k) O4-Fe0-S3

2.6.3 Torsional energy profiles

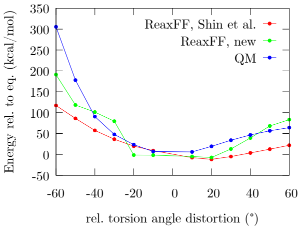

(a) S3-Fe1-S2-Fe0

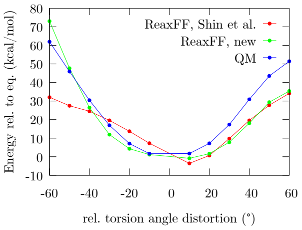

(b) Fe0-S2-Fe1-S3

2.6.4 Cluster-H_2O dissociation profiles

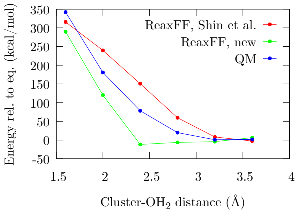

(a) Fe1-O13

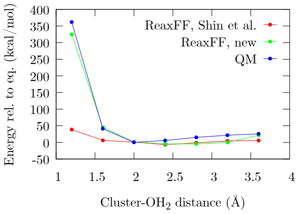

(b) Fe0-O4

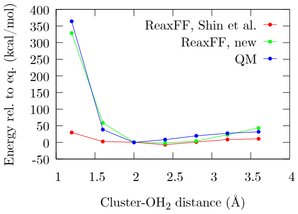

(c) Fe1-O10

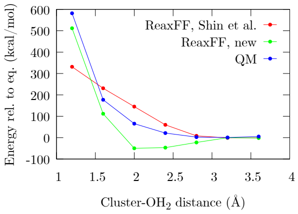

(d) Fe0-O7

2.7 $Fe_4S_4 + 4H_2O$

2.7.1 Dissociation profiles

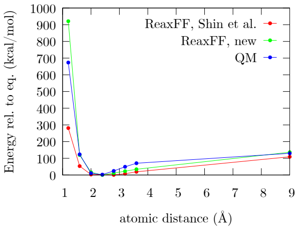

(a) S4-Fe0

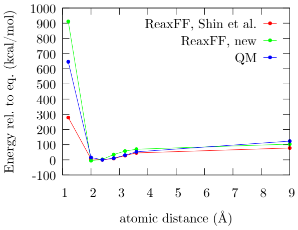

(b) Fe2-S6

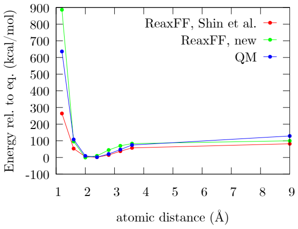

(c) Fe0-S4

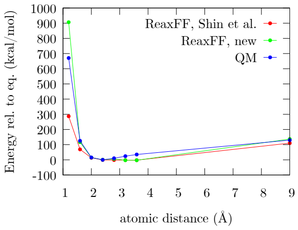

(d) S6-Fe2

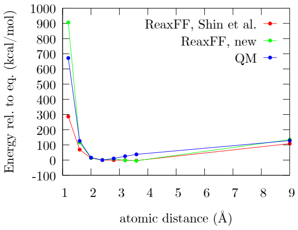

(e) S6-Fe0

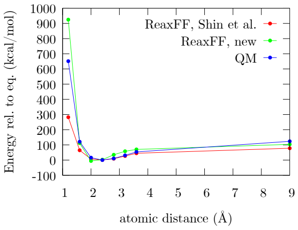

(f) Fe0S6

2.7.2 Angular energy profiles

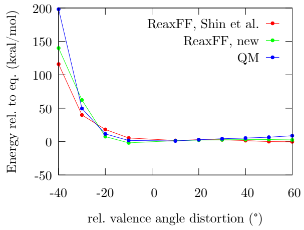

(a) S6-Fe0-O8

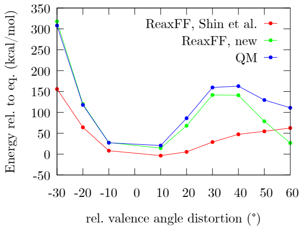

(b) Fe0-S5-Fe3

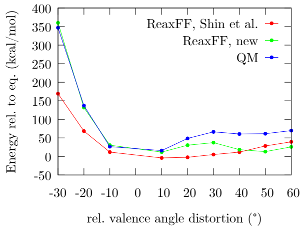

(c) Fe0-S4-Fe1

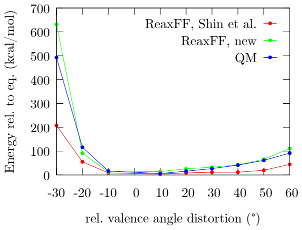

(d) S4-Fe0-S5

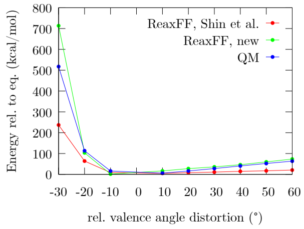

(e) S5-Fe3-S7

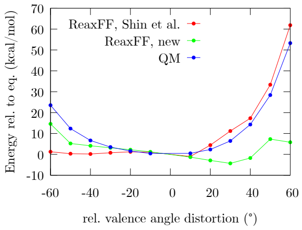

(f) S4-Fe0-O8

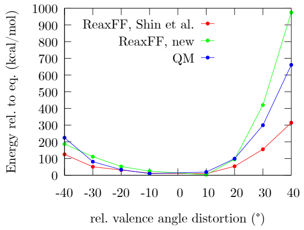

(g) O8-Fe0-S6

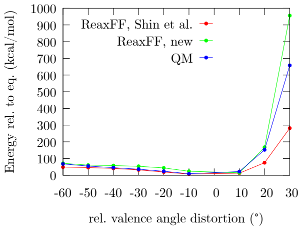

(h) O8-Fe0-S4

2.7.3 Torsional energy profiles

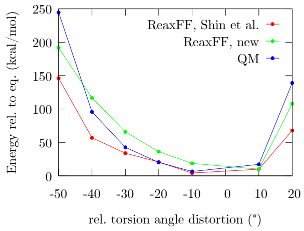

(a) Fe0-S5-Fe3-S7

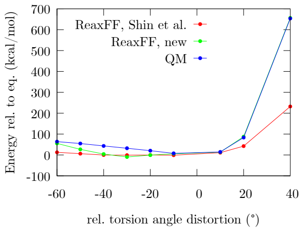

(b) S7-Fe1-S4-Fe0

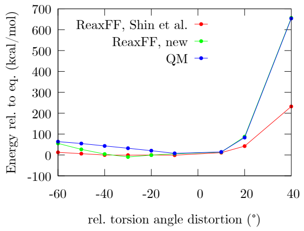

(c) S5-Fe1-S4-Fe0

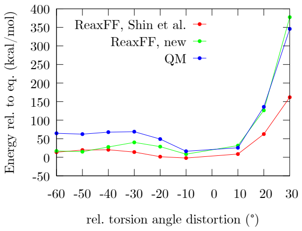

(d) S7-Fe3-S5-Fe0

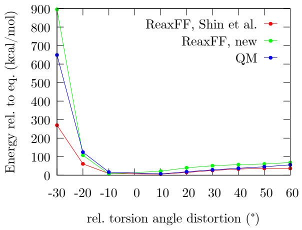

(e) Fe0-S4-Fe1-S5

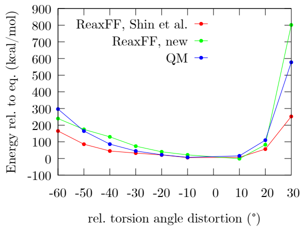

(f) Fe0-S4-Fe1-S7

2.7.4 Cluster-H_2O dissociation profiles

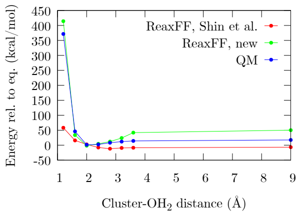

(a) Fe0-O8

```
ReaxFF ffield: aqueous FeS clusters (E. Moerman, D. Furman and D.J.Wales, 2020)
 39       ! Number of general parameters
   50.0000 !Overcoordination
    9.5469 !Overcoordination
    1.6725 !Valency
    1.7224 !Triple
    6.8702 !Triple
   60.4850 !C2-correction
    1.0588 !Undercoordination
    4.6000 !Triple
   12.1176 !Undercoordination
   13.3056 !Undercoordination
  -40.0000 !Triple
    0.0000 !Lower
   10.0000 !Upper
    2.8793 !Not
   33.8667 !Valency
    6.0891 !Valency
    1.0563 !Valency
    2.0384 !Valency
```

```
  6.1431 !Not
  6.9290 !Double
  0.3989 !Double
  3.9954 !Double
 -2.4837 !Not
  5.7796 !Torsion/BO
 10.0000 !Torsion
  1.9487 !Torsion
 -1.2327 !Conjugation
  2.1645 !Conjugation
  1.5591 !vdWaals
  0.1000 !Cutoff
  1.7602 !Valency
  0.6991 !Overcoordination
 50.0000 !Overcoordination
  1.8512 !Valency/lone
  0.5000 !Not
  0.0000 !Not
  5.0000 !Molecular
  0.0000 !Molecular
  0.7903 !Valency
 7    ! #atoms; atomID;ro(sigma); Val;atom mass;Rvdw;Dij;gamma;ro(pi);Val(e)
           alfa;gamma(w);Val(angle);p(ovun5);n.u.;chiEEM;etaEEM;n.u.
           ro(pipi);p(lp2);Heat increment;p(boc4);p(boc3);p(boc5),n.u.;n.u.
           p(ovun2);p(val3);n.u.;Val(boc);p(val5);n.u.;n.u.;n.u.
C    1.3817   4.0000  12.0000   1.8903   0.1838   0.6387   1.1341   4.0000
     9.7559   2.1346   4.0000  34.9350  79.5548   4.9218   6.0000   0.0000
```

	1.2114	0.0000	202.2908	8.9539	34.9289	13.5366	0.8563	0.0000
	-2.8983	2.5000	1.0564	4.0000	2.9663	2.0000	5.0000	3.0000
H	0.8930	1.0000	1.0080	1.3550	0.0930	0.5111	-0.1000	1.0000
	8.2230	33.2894	1.0000	0.0000	121.1250	-5.6808	12.1068	1.0000
	-0.1000	0.0000	55.1878	3.0408	2.4197	0.0003	1.0698	0.0000
	-19.4571	4.2733	1.0338	1.0000	2.8793	1.3913	0.0909	7.5146
O	1.6664	2.0000	15.9990	2.4075	0.1526	0.7593	1.5072	6.0000
	6.9465	26.6793	4.0000	19.6565	116.0768	-0.3647	6.6691	2.0000
	0.5298	9.9305	121.7005	8.4190	32.9723	6.0767	0.9745	0.0000
	-2.5630	2.5435	1.0493	4.0000	5.9671	1.0678	0.1303	5.6842
N	1.2333	3.0000	14.0000	2.1294	0.1322	1.0000	1.1748	5.0000
	10.0056	10.8657	4.0000	30.9146	100.0000	6.4603	7.0317	2.0000
	1.0433	4.7941	119.9837	0.6005	7.9731	2.2800	0.9745	0.0000
	-4.6366	4.0000	1.0183	4.0000	2.8793	4.0000	4.0000	4.0000
S	0.9768	2.0000	32.0600	1.3707	0.5574	1.0146	1.4108	6.0000
	13.1888	63.0522	4.0000	22.3105	112.1416	6.3379	11.0224	2.0000
	0.3208	30.7861	145.2260	0.2100	80.0000	0.0000	0.9745	0.0000
	-2.5145	4.3114	1.0338	6.2998	4.9638	0.9080	0.0624	6.7627
Fe	2.1537	3.0000	55.8450	1.5201	0.6524	0.8149	0.0723	3.0000
	11.2885	64.8872	3.0000	-4.2360	0.0000	-6.8364	13.8922	0.0000
	0.2152	0.0000	218.5839	140.0000	80.0000	0.0000	0.0000	0.0000
	-1.2779	1.1563	1.0564	6.0000	2.6089	1.4626	0.1200	14.9819
X	-0.0998	2.0000	1.0080	2.0000	0.0000	1.0000	-0.1000	6.0000
	10.0000	2.5000	4.0000	0.0000	0.0000	8.5000	1.5000	0.0000
	-0.1000	0.0000	-2.3700	8.7410	13.3640	0.6690	0.9745	0.0000
	-11.0000	2.7466	1.0338	4.0000	2.8793	3.0000	4.0000	4.0000

21 ! #bonds;at1;at2;De(sig);De(pi);De(pipi);p(be1);p(bo5);13cor;n.u.;p(bo6),p(ovun1)

p(be2);p(bo3);p(bo4);n.u.;p(bo1);p(bo2)

1	1	158.2004	99.1897	78.0000	-0.7738	-0.4550	1.0000	37.6117	0.4147
		0.4590	-0.1000	9.1628	1.0000	-0.0777	6.7268	1.0000	0.0000
1	2	169.4760	0.0000	0.0000	-0.6083	0.0000	1.0000	6.0000	0.7652
		5.2290	1.0000	0.0000	1.0000	-0.0553	6.9316	0.0000	0.0000
2	2	153.3934	0.0000	0.0000	-0.4600	0.0000	1.0000	6.0000	0.7300
		6.2500	1.0000	0.0000	1.0000	-0.0790	6.0552	0.0000	0.0000
1	3	115.3161	127.1562	61.7072	-0.5141	-0.3474	1.0000	18.9948	0.9954
		1.5618	-0.3414	8.9489	1.0000	-0.1628	5.6821	0.0000	0.0000
3	3	113.0720	163.1074	86.8283	-0.5208	-0.8248	1.0000	16.8428	0.3861
		9.5726	-0.3608	11.0392	1.0000	-0.4290	13.5706	1.0000	0.0000
1	4	164.1304	141.3380	102.0464	-1.8021	-0.5696	1.0000	27.6095	0.2487
		0.3953	-0.3663	7.1330	1.0000	-0.2557	4.6940	1.0000	0.0000
3	4	128.8596	167.8643	40.0000	-0.3819	-0.1539	1.0000	34.9972	0.1900
		1.0110	-0.3716	7.0805	1.0000	-0.1265	6.8843	1.0000	0.0000
4	4	160.1592	82.5526	153.9884	-0.4110	-0.0934	1.0000	12.4304	0.5899
		0.1538	-0.1473	11.9187	1.0000	-0.0753	5.4371	1.0000	0.0000
2	3	300.0000	0.0000	0.0000	-0.6540	0.0000	1.0000	6.0000	0.1489
		8.6930	1.0000	0.0000	0.0000	-0.1916	6.6828	0.0000	0.0000
2	4	211.6032	0.0000	0.0000	-0.3415	0.0000	1.0000	6.0000	0.4726
		2.7198	1.0000	0.0000	1.0000	-0.1744	5.6399	0.0000	0.0000
1	5	150.8132	59.3363	55.2528	-0.0628	-0.5211	1.0000	18.9617	0.3219
		0.3317	-0.2289	7.5946	1.0000	-0.1946	5.9455	1.0000	0.0000
2	5	143.4377	0.0000	0.0000	-0.2944	0.0000	1.0000	6.0000	0.6034
		9.5627	1.0000	0.0000	1.0000	-0.0516	7.0960	1.0000	0.0000
3	5	107.2917	202.9813	40.0000	-0.5563	-0.4038	1.0000	49.5611	0.6000
		0.4259	-0.4577	12.7569	1.0000	-0.1100	7.1145	1.0000	0.0000

```
  4  5   0.0000   0.0000   0.0000  -0.4438  -0.2034   1.0000  40.3399   0.6000
         0.3296  -0.3153   9.1227   1.0000  -0.1805   5.6864   1.0000   0.0000
  5  5 300.0000  92.6426 180.3003  -1.5000  -0.1312   1.0000   2.5945  -0.1500
        21.0000  -0.2616   3.8082   1.0000  -0.1191  14.2113   5.7663  15.7724
  1  6  58.6896   0.0000   0.0000  -0.0203  -0.1418   1.0000  13.1260   0.0230
         8.2136  -0.1310   0.0000   1.0000  -0.2692   6.4254   0.0000  24.4461
  2  6  58.6896   0.0000   0.0000  -0.0203  -0.1418   1.0000  13.1260   0.0230
         8.2136  -0.1310   0.0000   1.0000  -0.2692   6.4254   0.0000  24.4461
  3  6  83.4356   0.0000   0.0000  -0.0622  -0.8066   1.0000   5.9363  -0.0361
         1.2260  -0.8066   5.9363   1.0000  -0.0128  11.2876   3.7659   4.3454
  4  6  87.0227   0.0000  43.3991  -0.0030  -0.3000   1.0000  36.0000   0.0250
         0.0087  -0.2500  12.0000   1.0000  -0.0439   6.6073   1.0000  24.4461
  5  6 106.2620  97.6981 174.6504  -0.0022  -0.6755   1.0000   8.6354  -0.0188
         9.7068  -0.4429  29.0329   1.0000  -0.2394   4.7860   2.2267  13.5408
  6  6  27.6789 168.5947 188.4954  -1.4666  -0.2688   0.0000  18.5814   0.2844
        20.9974  -0.5562   0.9513   1.0000  -0.1097   8.0367   4.3853   0.0000
 13    ! #off-diagonal terms. at1;at2;Dij;RvdW;alfa;ro(sigma);ro(pi);ro(pipi)
  1  2   0.1239   1.4004   9.8467   1.1210  -1.0000  -1.0000
  2  3   0.1242   1.7487   9.4791   1.2365   1.2845   1.3274
  2  4   0.1664   1.3100   9.6406   1.0569  -1.0000  -1.0000
  1  3   0.0503   1.8006  10.2114   1.3492   1.1992   1.0506
  1  4   0.1771   1.8995   9.6891   1.3428   1.2492   1.1154
  3  4   0.2000   1.8388   9.5137   1.4587   1.0933   1.1826
  1  5   0.1618   1.7943  10.1042   1.7489   1.3150   1.4031
  2  5   0.0764   1.5838  10.1462   1.4206  -1.0000  -1.0000
  3  5   0.1022   1.9887  10.0605   1.5799   1.4000  -1.0000
  4  5   0.1505   1.9000  10.5104   1.8000   1.4000  -1.0000
```

```
 2  6   0.0100   1.6000  13.2979   1.8670  -1.0000  -1.0000
 3  6   0.2220   1.5790  12.0514   2.2896   2.2896   1.0930
 5  6   0.3635   1.6323  12.6111   1.6620   0.9724   1.5194
76     ! #angles.at1;at2;at3;Thetao,o;p(val1);p(val2);p(coa1);p(val7);p(pen1);p(val4)
 1  1  1  59.0573  30.7029   0.7606   0.0000   0.7180   6.2933   1.1244
 1  1  2  65.7758  14.5234   6.2481   0.0000   0.5665   0.0000   1.6255
 2  1  2  70.2607  25.2202   3.7312   0.0000   0.0050   0.0000   2.7500
 1  2  2   0.0000   0.0000   6.0000   0.0000   0.0000   0.0000   1.0400
 1  2  1   0.0000   3.4110   7.7350   0.0000   0.0000   0.0000   1.0400
 2  2  2   0.0000  27.9213   5.8635   0.0000   0.0000   0.0000   1.0400
 1  1  3  54.7427  21.1992   1.0613   0.0000   2.9950  58.6562   1.1232
 3  1  3  78.6632  16.3065   6.3613 -19.9300   1.5183   0.0000   2.2234
 1  1  4  78.9895  29.7448   1.4146   0.0000   1.1834   0.0000   2.4298
 3  1  4  74.5431  30.9283   1.2618   0.0000   1.1019   0.0000   1.0888
 4  1  4  90.0000  15.9388   0.5081   0.0000   1.1155   0.0000   2.5891
 2  1  3  50.0000  12.9103   2.5311   0.0000   0.1000   0.0000   1.0000
 2  1  4  73.8008  28.9565   1.9450   0.0000   0.2000   0.0000   2.9066
 1  2  4   0.0000   0.0019   6.3000   0.0000   0.0000   0.0000   1.0400
 1  3  1  71.6401  45.0000   1.2667   0.0000   2.8294   0.0000   1.0000
 1  3  3  76.3686  44.8665   1.9461   0.0000   1.0572  68.1072   1.8676
 1  3  4  70.4701  35.0124   2.2286   0.0000   2.9000   0.0000   2.4754
 3  3  3  89.9293  15.8855   2.0229   0.0000   2.9881   0.0000   1.0237
 3  3  4  84.0202  31.3592   1.0534   0.0000   2.9000   0.0000   1.4406
 4  3  4  72.3904  15.0722   5.0227   0.0000   3.0072   0.0000   1.0000
 1  3  2  90.0000   6.6459   5.2255   0.0000   1.3111   0.0000   3.0000
 2  3  3  81.0907  50.0000   2.0000   0.0000   1.0000   0.0000   1.5863
 2  3  4  68.4187  33.4407   7.5000   0.0000   0.1000   0.0000   1.0000
```

2	3	2	85.8000	9.8453	2.2720	0.0000	2.8635	0.0000	1.5800
1	4	1	81.2266	17.5379	1.2324	0.0000	2.8702	0.0000	1.0000
1	4	3	73.8735	39.1639	1.0445	0.0000	2.8701	0.0000	1.7008
1	4	4	71.3629	18.4874	2.3468	0.0000	2.8701	0.0000	1.8255
3	4	3	74.9086	21.9109	2.5904	-18.0069	3.0701	0.0000	1.0000
3	4	4	77.8757	28.9944	1.2740	-0.9193	3.0117	0.0000	1.0000
4	4	4	76.1795	29.2290	1.6529	0.0000	2.9983	0.0000	2.4525
1	4	2	69.0828	11.0941	2.4635	0.0000	0.2025	0.0000	2.3768
2	4	3	77.7697	23.7768	2.7987	0.0000	0.3956	0.0000	3.0000
2	4	4	74.3012	42.0419	1.2591	0.0000	0.5437	0.0000	1.1369
2	4	2	84.3282	13.8208	4.6573	0.0000	0.1000	0.0000	1.0000
1	2	3	0.0000	16.7302	1.1143	0.0000	0.0000	0.0000	1.0000
1	2	4	0.0000	14.7285	3.8173	0.0000	0.0000	0.0000	2.1043
1	2	5	0.0000	15.0000	3.0000	0.0000	0.0000	0.0000	1.0400
3	2	3	0.0000	15.0000	2.8900	0.0000	0.0000	0.0000	2.8774
3	2	4	0.0000	1.4986	0.1000	0.0000	0.0000	0.0000	3.0000
4	2	4	0.0000	2.4033	0.1000	0.0000	0.0000	0.0000	1.8653
2	2	3	0.0000	8.5744	3.0000	0.0000	0.0000	0.0000	1.0421
2	2	4	0.0000	0.0019	6.0000	0.0000	0.0000	0.0000	1.0400
1	1	5	74.4180	33.4273	1.7018	0.1463	0.5000	0.0000	1.6178
1	1	6	74.4180	33.4273	1.7018	0.1463	0.5000	0.0000	1.6178
1	5	1	79.7037	28.2036	1.7073	0.1463	0.5000	0.0000	1.6453
2	1	5	63.3289	29.4225	2.1326	0.0000	0.5000	0.0000	3.0000
1	5	2	85.9449	38.3109	1.2492	0.0000	0.5000	0.0000	1.1000
1	5	5	80.0000	25.0000	2.0000	0.0000	0.5000	0.0000	1.3830
2	5	2	85.0000	15.1317	2.0000	0.0000	0.5000	0.0000	2.0000
2	5	5	97.0064	32.1121	2.0242	0.0000	0.5000	0.0000	2.8568

2	2	5	0.0000	0.0019	6.0000	0.0000	0.0000	0.0000	1.0400
2	2	6	97.0064	32.1121	2.0242	0.0000	0.5000	0.0000	2.8568
5	4	5	62.0000	33.4273	1.7018	0.1463	0.5000	0.0000	1.0500
3	5	3	77.0699	39.4349	2.1313	-30.0000	0.9567	0.0000	1.1483
1	5	3	70.0000	35.0000	3.4223	0.0000	1.3550	0.0000	1.2002
1	5	4	70.0000	35.0000	3.4223	0.0000	1.3550	0.0000	1.2002
3	5	4	70.0000	35.0000	3.4223	0.0000	1.3550	0.0000	1.2002
1	3	5	73.0990	33.8942	1.2098	0.0000	0.8161	0.0000	1.1776
3	3	5	83.9753	31.0715	3.5590	0.0000	0.8161	0.0000	1.1776
2	3	5	76.9521	20.0000	2.0903	0.0000	1.0000	0.0000	1.0400
2	6	2	0.0000	49.8261	0.2093	0.0000	2.0870	0.0000	2.2895
2	2	6	0.0000	39.7818	3.1505	0.0000	1.1296	0.0000	1.1110
6	2	6	0.0000	0.5047	0.8000	0.0000	0.8933	0.0000	4.6650
2	6	6	0.0000	8.7037	0.0827	0.0000	3.5597	0.0000	1.1198
3	6	3	123.8785	57.6570	23.2531	-11.4673	2.7737	0.0000	1.7782
6	3	6	0.0008	25.0000	8.0000	0.0000	1.0000	0.0000	3.0000
2	3	6	66.0423	5.0000	1.0000	0.0000	1.0000	0.0000	1.2500
2	6	3	0.0000	0.5000	0.1000	0.0000	1.0000	0.0000	3.0000
3	3	6	70.0000	20.0000	1.0000	0.0000	1.0000	0.0000	1.2500
5	6	5	70.1226	12.8890	23.8755	-42.6716	4.8077	0.0000	1.7263
5	5	6	125.3731	-15.7260	26.0741	-3.8073	0.3023	0.0000	0.8430
6	6	5	36.9927	11.4717	19.8822	-6.5576	4.3398	0.0000	3.7892
6	3	3	70.0000	20.0000	1.0000	0.0000	1.0000	0.0000	1.2500
6	5	6	66.9222	-1.2888	29.8567	-36.4256	1.2982	0.0000	1.3233
6	6	3	7.3389	-27.9740	0.0505	-26.4436	-0.1154	0.0000	1.4947
5	6	3	-0.4625	-35.2861	19.7744	-28.5471	3.9041	0.0000	1.0947

48 ! #torsions. at1;at2;at3;at4;;V1;V2;V3;p(tor1);p(cot1);n.u;n.u.

1	1	1	1	-0.2500	34.7453	0.0288	-6.3507	-1.6000	0.0000	0.0000
1	1	1	2	-0.2500	29.2131	0.2945	-4.9581	-2.1802	0.0000	0.0000
2	1	1	2	-0.2500	31.2081	0.4539	-4.8923	-2.2677	0.0000	0.0000
1	1	1	3	-2.5000	25.4016	1.0000	-4.4850	-1.1000	0.0000	0.0000
2	1	1	3	-0.9763	59.4161	1.0000	-7.7414	-1.0978	0.0000	0.0000
3	1	1	3	-2.5000	52.7614	-1.0000	-4.0134	-0.8614	0.0000	0.0000
1	1	3	1	-1.9125	80.0000	-1.0000	-4.5626	-0.9000	0.0000	0.0000
1	1	3	2	0.6154	8.3019	-0.4870	-2.9336	-0.9000	0.0000	0.0000
2	1	3	1	-2.5000	80.0000	0.9658	-4.4935	-0.9000	0.0000	0.0000
2	1	3	2	-1.0000	31.8695	1.0000	-2.6151	-1.1000	0.0000	0.0000
1	1	3	3	0.7514	34.1941	0.5669	-5.5360	-2.0544	0.0000	0.0000
2	1	3	3	2.5000	80.0000	1.0000	-2.6841	-2.8274	0.0000	0.0000
3	1	3	1	0.2515	79.1495	-0.6263	-4.3647	-3.0437	0.0000	0.0000
3	1	3	2	1.0000	37.1243	1.0000	-2.5000	-3.0476	0.0000	0.0000
3	1	3	3	-1.0092	41.0504	0.3915	-6.0913	-2.7174	0.0000	0.0000
1	3	3	1	-1.6378	-11.8357	0.3815	-3.2104	-2.7536	0.0000	0.0000
1	3	3	2	-2.5000	-9.2805	0.3063	-5.9187	-2.9498	0.0000	0.0000
2	3	3	2	0.2732	-21.6925	-1.0000	-2.5000	-0.9921	0.0000	0.0000
1	3	3	3	2.5000	-17.6041	1.0000	-2.5000	-0.9972	0.0000	0.0000
2	3	3	3	-2.5000	78.0855	-0.8750	-7.8902	-1.2407	0.0000	0.0000
3	3	3	3	-2.5000	-25.0000	1.0000	-2.5000	-0.9000	0.0000	0.0000
1	1	4	2	1.4427	31.7903	0.2054	-8.0000	-1.9825	0.0000	0.0000
2	1	4	2	-1.0000	64.2008	0.3037	-7.5233	-2.1051	0.0000	0.0000
3	1	4	2	1.0000	29.1410	1.0000	-3.2244	-2.5261	0.0000	0.0000
3	1	1	4	-1.0000	65.1457	0.2433	-4.9542	-0.9511	0.0000	0.0000
4	1	1	4	1.0000	87.8413	0.3817	-3.7479	-1.7241	0.0000	0.0000
1	1	4	1	1.0000	12.2873	0.7438	-3.5510	-1.6589	0.0000	0.0000

```
 3  1  4  1  -1.0000  -0.2183   1.0000  -3.5014  -1.8038   0.0000   0.0000
 2  1  1  4   1.0000  23.7736   0.4235  -2.7665  -1.9000   0.0000   0.0000
 4  1  4  2   1.0000  96.1436   1.0000  -6.9528  -2.0202   0.0000   0.0000
 2  1  4  1  -1.0000  88.5527  -0.3433  -7.9806  -1.5996   0.0000   0.0000
 0  1  2  0   0.0000   0.0000   0.0000   0.0000   0.0000   0.0000   0.0000
 0  2  2  0   0.0000   0.0000   0.0000   0.0000   0.0000   0.0000   0.0000
 0  2  3  0   0.0000   0.1000   0.0200  -2.5415   0.0000   0.0000   0.0000
 0  1  1  0   0.0000  50.0000   0.3000  -4.0000  -2.0000   0.0000   0.0000
 0  3  3  0   0.5511  25.4150   1.1330  -5.1903  -1.0000   0.0000   0.0000
 0  1  4  0   0.2176  40.4126   0.3535  -3.9875  -2.0051   0.0000   0.0000
 0  2  4  0   0.0000   0.1032   0.3000  -5.0965   0.0000   0.0000   0.0000
 0  3  4  0   1.1397  61.3225   0.5139  -3.8507  -2.7831   0.0000   0.0000
 0  4  4  0   0.7265  44.3155   1.0000  -4.4046  -2.0000   0.0000   0.0000
 4  1  4  4  -0.0949   8.7582   0.3310  -7.9430  -2.0000   0.0000   0.0000
 0  1  5  0   0.8251  92.1468   0.7176  -4.2341   0.0000   0.0000   0.0000
 0  2  5  0   0.0000   0.0000   0.0000   0.0000   0.0000   0.0000   0.0000
 0  2  6  0   0.0000   0.0000   0.1200  -2.4847   0.0000   0.0000   0.0000
 0  3  6  0   0.0000   0.0000   0.1200  -2.4703   0.0000   0.0000   0.0000
 1  1  3  3  -0.0002  20.1851   0.1601  -9.0000  -2.0000   0.0000   0.0000
 1  3  3  1   0.0002  80.0000  -1.5000  -4.4848  -2.0000   0.0000   0.0000
 3  1  3  3  -0.1583  20.0000   1.5000  -9.0000  -2.0000   0.0000   0.0000
 9    ! #hydrogen bonds. at1;at2;at3;r(hb);p(hb1);p(hb2);p(hb3)
 3  2  3   1.4395  -2.7649   4.2575  22.8634
 3  2  4   2.0985  -4.5000   1.4500  19.5000
 4  2  3   1.7500  -1.5000   1.4500  19.5000
 4  2  4   1.9893  -3.2987   1.4500  19.5000
 3  2  5   1.8500 -12.0000   5.0000   5.4510
```

4	2	5	1.5000	-2.0000	1.4500	19.5000
5	2	3	1.5000	-2.0000	1.4500	19.5000
5	2	4	1.5000	-2.0000	1.4500	19.5000
5	2	5	1.5000	-2.0000	1.4500	19.5000
